# Improving Executive Functioning in Children with ADHD: Training Multiple Executive Functions within the Context of a Computer Game. A Randomized Double-Blind Placebo Controlled Trial

**DOI:** 10.1371/journal.pone.0121651

**Published:** 2015-04-06

**Authors:** Sebastiaan Dovis, Saskia Van der Oord, Reinout W. Wiers, Pier J. M. Prins

**Affiliations:** 1 Department of Developmental Psychology, University of Amsterdam, Amsterdam, The Netherlands; 2 Addiction, Development, and Psychopathology (Adapt) Lab, Department of Psychology, University of Amsterdam, Amsterdam, The Netherlands; 3 Cognitive Science Center Amsterdam, University of Amsterdam, Amsterdam, The Netherlands; 4 Department of Clinical Psychology, Leuven University, Leuven, Belgium; Monash University, AUSTRALIA

## Abstract

**Introduction:**

Executive functions (EFs) training interventions aimed at ADHD-symptom reduction have yielded mixed results. Generally, these interventions focus on training a single cognitive domain (e.g., working memory [WM], inhibition, or cognitive-flexibility). However, evidence suggests that most children with ADHD show deficits on multiple EFs, and that these EFs are largely related to different brain regions. Therefore, training multiple EFs might be a potentially more effective strategy to reduce EF-related ADHD symptoms.

**Methods:**

Eighty-nine children with a clinical diagnosis of ADHD (aged 8–12) were randomized to either a *full-active-condition* where visuospatial WM, inhibition and cognitive-flexibility were trained, a *partially-active-condition* where inhibition and cognitive-flexibility were trained and the WM-training task was presented in placebo-mode, or to a full *placebo-condition*. Short-term and long-term (3-months) effects of this *gamified*, 25-session, home-based computer-training were evaluated on multiple outcome domains.

**Results:**

During training compliance was high (only 3% failed to meet compliance criteria). After training, only children in the full-active condition showed improvement on measures of visuospatial short-term-memory (STM) and WM. Inhibitory performance and interference control only improved in the full-active- and the partially-active condition. No Treatment-condition x Time interactions were found for cognitive-flexibility, verbal WM, complex-reasoning, nor for any parent-, teacher-, or child-rated ADHD behaviors, EF-behaviors, motivational behaviors, or general problem behaviors. Nonetheless, almost all measures showed main Time-effects, including the teacher-ratings.

**Conclusions:**

Improvements on inhibition and visuospatial STM and WM were specifically related to the type of treatment received. However, transfer to untrained EFs and behaviors was mostly nonspecific (i.e., only interference control improved exclusively in the two EF training conditions). As such, in this multiple EF-training, mainly nonspecific treatment factors – as opposed to the specific effects of training EFs—seem related to far transfer effects found on EF and behavior.

**Trial Registration:**

trialregister.nl NTR2728. Registry name: improving executive functioning in children with ADHD: training executive functions within the context of a computer game; registry number: NTR2728.

## Introduction

Theories of ADHD suggest that deficits in executive functioning are at the core of the ADHD-syndrome, and play a pivotal role in explaining the problems children with ADHD encounter in daily life [1], [2], [3], [4]. Via dorsal frontostriatal brain circuits, executive functions (EF) allow individuals to regulate their behavior, thoughts and emotions, and thereby enable self-control [5]. Evidence indeed suggests that impairments in EF are related to deficits in attention, hyperactivity and impulsivity [6], [7], [8], [9], [10], [11], and with associated problems such as deficient academic functioning [12], [13]. Moreover, research suggests that EF-capacity and its associated levels of brain activity are not static, but may be altered by task-repetition or training [14]. Therefore, in the past few years, EF training interventions aimed at ADHD symptom reduction have received considerable interest.

Nonetheless, these EF interventions have yielded mixed results, especially on ADHD behavior (for an overview see [15], [16], [17], [18], [19], [20]; in addition see [21], [22], [23]). Generally, these interventions focus on training a single domain of cognitive functioning in children with ADHD, such as working memory (WM), inhibition, or cognitive flexibility. However, evidence suggests that most children with ADHD show deficits on multiple EFs [24], and that these EFs are largely related to different brain regions [25], [26], [27]. Therefore, training of multiple EFs might be a potentially more effective strategy to reduce EF related ADHD symptoms.

To date, evidence for multiple EF training interventions is limited. Few studies have investigated the effects of these interventions in children with ADHD [28], [29], [30], [31], [32], [33], and although these studies generally show promising results (e.g., improvement of ADHD behavior as rated by parents and/or a significant other [e.g., the teacher]; an increase of neural activity and gray matter volume in ADHD affected brain areas), none of these studies are placebo-controlled.

Besides EF deficits, children with ADHD have problems with motivation. Motivational models [34], [35], [36], [37], and subsequent research (for an overview see [38], [39]; also see [40], [41], [42], [43]) suggest that children with ADHD are less stimulated by reinforcement (i.e. reward) than typically developing children (probably due to a dopaminergic deficit), and therefore require higher amounts and frequencies of reward in order to perform optimally. This elevated need for reinforcement in children with ADHD may result in motivational problems during EF training: the child has to repeat the same responses over and over again for many trials, making most EF training programs tedious and boring for children with ADHD [44]. Research suggests that motivational problems can decrease the effects of EF training in children with ADHD [45]. However, gamification of an EF training or task (e.g., by using game mechanics and visuals) has been found to optimize both motivation and training-effects in children with ADHD [40], [45], [46]. Gaming increases the release of striatal dopamine [47], [48], promoting long-term potentiation of neural connections within the striatum [49], which is suggested to improve motivation and one’s ability to learn [50] (e.g., during EF training).

In the current double-blind, placebo-controlled study, we investigated the efficacy of a gamified, 5-week, home-based, multiple EF training intervention titled Braingame Brian (BGB; [44]) in children with ADHD (combined-subtype). A previous waitlist-controlled study of BGB [31] showed promising results on reduction of symptoms of ADHD and improvement of EF. BGB targets multiple EFs that are commonly impaired in children with ADHD: visuospatial WM, response inhibition, and cognitive flexibility [51]. To date, most EF-training studies focus on the effects of WM training (e.g., see [15]), whereas very few studies investigate the unique effects (i.e. without WM training) of response inhibition- and/or cognitive flexibility training in children with ADHD. Only Kray et al. [23] investigated effects of a cognitive flexibility training in children with ADHD; they found placebo-controlled effects on untrained EF performance (i.e., interference control), but they did not investigate effects on behavior. Moreover, we are not aware of any studies investigating the unique effects of inhibition training in children with ADHD (for studies of combined WM and inhibition training see [28], [29], [30]). Therefore, participants in the current study were randomized to one of three treatment conditions: (1) a full-active-condition where visuospatial WM, response inhibition and cognitive flexibility were trained, (2) a partially-active-condition where only inhibition and cognitive flexibility were trained and the visuospatial WM training-task was presented in placebo-mode, or (3) to a full placebo-condition. Short-term and long-term (3-months) effects were evaluated across various outcome measures (including performance measures of WM, inhibition, cognitive-flexibility, interference control, and complex reasoning, and rating scales assessing parent- and teacher-rated ADHD behavior, parent-rated EF- and motivational behavior, and parent-, teacher- and child-rated general problem behavior).

We expected that: (1) improvement on outcome measures of WM, inhibition, and cognitive flexibility (i.e., performance measures and EF rating-scales) would be specifically related to the type of treatment received (e.g., greatest improvement on WM if WM was trained), (2) the (far-) transfer of treatment effects to other, untrained, domains of EF (such as interference control or parent-rated planning, organization of materials or self-monitoring) would be limited. We expected that spill-over effects to untrained domains of EF (far transfer) would be limited because different EFs are largely related to different brain regions [25], [26], [27], and because most placebo-controlled EF training studies that investigate children with ADHD do not find such far transfer effects (e.g., see [19]), (3) children in the full-active condition would improve significantly more on ADHD behavior than children in either the partially-active condition or placebo condition, and (4) children in the partially-active condition would improve significantly more on ADHD behavior than children in the placebo condition. Finally, we also investigated other domains of impairment that are associated with ADHD (such as sensitivity to reward and punishment, oppositional defiant behavior, quality of life, and problems in daily situations). However, given the current knowledge-base in the field (e.g., there are no placebo-controlled EF training studies that investigate effects on sensitivity to reward and punishment, quality of life or problems in daily situations, and placebo-controlled studies investigating effects on oppositional defiant behavior show mixed results [21], [23], [84], [85], [86]), we refrained from presenting hypotheses regarding these domains of impairment.

## Methods

### Trial Design

This was a multicenter (14 sites), double-blind, placebo-controlled, multi-arm parallel-group study conducted in the Netherlands (trial register: http://www.trialregister.nl/trialreg/admin/rctview.asp?TC=2728; registry name: improving executive functioning in children with ADHD: training executive functions within the context of a computer game; registry number: NTR2728). No important changes to methods were made after trial commencement (the trial started April 2011 and ended January 2013). The protocol for this trial and CONSORT checklist are available as S1 Protocol and S1 CONSORT Checklist.

### Participants

#### Study settings

Children were recruited from 14 outpatient mental-healthcare centers. This study was conducted in the Netherlands, within a predominantly urban type of community.

#### Eligibility criteria

Eligible participants were all children aged 8 to 12 years with (a) a prior DSM-IV-TR [52] diagnosis of ADHD combined-type and absence of any autism spectrum disorder according to a child psychologist or psychiatrist, (b) a score within the clinical range (95^th^ to 100^th^ percentile) on the ADHD scales of both the parent and teacher version of the Disruptive Behavior Disorder Rating Scale (DBDRS [53]; Dutch translation: [54]), (c) meeting criteria for ADHD combined-type on the ADHD section of the Diagnostic Interview Schedule for Children, parent version (PDISC-IV [55]). The PDISC-IV is a structured diagnostic interview based on the DSM-IV, with adequate psychometric properties, (d) absence of conduct disorder (CD) based on the CD sections of the PDISC-IV, (e) an IQ score ≥80 established by the short version of the Dutch Wechsler Intelligence Scale for Children (WISC-III; [56]). Two subtests, Vocabulary and Block Design, were administered to estimate Full Scale IQ (FSIQ). This composite score has satisfactory reliability and correlates highly with FSIQ [57], (f) absence of any neurological disorder, sensory (color blindness, vision) or motor impairment as stated by the parents, (g) not taking any medication other than Methylphenidate or Dextroamphetamine. Participants discontinued their Methylphenidate at least 24 hours before each test-session, allowing a complete wash-out [58]. Participants taking Dextroamphetamine discontinued medication 48 hours before each test-session [59], finally, (h) parents had to agree to keep the dose of ADHD medication stable between the intake and the 3-months follow-up session, and had to consent not to initiate or participate in other psychosocial treatments. Group differences in baseline demographics and clinical characteristics are listed in Table 1.

**Table 1:** Baseline Demographics and Clinical Characteristics by Treatment Group.

Measure	Treatment Group							
	Full-Active		Partially-Active		Placebo			
	(n = 31)		(n = 28)		(n = 30)			
	M	SD	M	SD	M	SD	F / χ^2^	*Group Comparison* ^*a*^
Gender (M:F)	25:6	-	22:6	-	24:6	-	.04	ns (*p* = .980)
Age (years)	10.6	1.4	10.3	1.3	10.5	1.3	.58	ns (*p* = .564)
FSIQ	101	11.5	101	11.4	101	11.6	.05	ns (*p* = .956)
*DBDRS parent*								
Inattention	22.0	3.6	21.3	4.1	21.9	4.6	.23	ns (*p* = .793)
Hyperactivity/Impulsivity	21.3	3.8	20.0	4.6	20.5	5.1	.69	ns (*p* = .504)
ODD	11.6	5.8	12.8	4.6	11.7	5.9	.40	ns (*p* = .674)
CD	2.9	3.1	2.7	2.9	3.2	2.9	.20	ns (*p* = .820)
*DBDRS teacher*								
Inattention	16.1	5.6	15.9	5.0	18.0	4.8	1.54	ns (*p* = .220)
Hyperactivity/Impulsivity	13.8	6.2	14.3	5.8	16.6	6.0	1.84	ns (*p* = .166)
ODD	7.4	6.0	7.1	5.0	8.6	6.6	.49	ns (*p* = .614)
CD	1.1	1.7	2.1	3.0	1.9	2.5	1.22	ns (*p* = .300)
*PDISC-IV*								
ODD diagnosis, *N* (%)	17 (55%)	-	18 (64%)	-	15 (50%)	-	1.24	ns (*p* = .539)
ADHD medication^b^, *N* (%)	20 (65%)	-	19 (68%)	-	22 (73%)	-	.56	ns (*p* = .756)
Computergame experience (hours per week)	8.6	5.0	9.8	9.1	11.6	8.4	1.17	ns (*p* = .314)
Dyscalculia, *N* (%)	0 (0%)	-	0 (0%)	-	0 (0%)	-	-	-
Dyslexia, *N* (%)	2 (7%)	-	5 (18%)	-	5 (17%)	-	2.03	ns (*p* = .362)

*Note*. CD = conduct disorder; DBDRS = Disruptive Behavior Disorder Rating Scale; FSIQ = full scale IQ; M:F = Male:Female; ODD = oppositional defiant disorder; PDISC-IV = Diagnostic Interview Schedule for Children, parent version;

^a^ Continuous data were investigated using ANOVAs. Nominal data were investigated using Pearson's chi-squared tests;

^b^ Four children were taking Dextroamphetamine (two in the full-active condition, one in the partially-active condition, and one in the placebo condition).

### Treatment Conditions

#### General characteristics of the intervention

“Braingame Brian” (BGB [44]) is a computerized, home-based EF training, embedded in a game world and is named after its main character “Brian”. Brian is a young inventor who, throughout the game, helps and befriends the game-worlds inhabitants by creating increasingly elaborate inventions (e.g., a delivery-rocket for the grocery-store owner). BGB consists of 25 training sessions. Within each session, the player can create inventions by completing two *blocks* of three training tasks. Within each block, the first training task is always a WM task (used for drawing a blueprint of the invention), the second and third task, a cognitive flexibility task and an inhibition task, are presented in changing order (and are used for sorting building-materials, and electrically-charging the invention). Each session takes about 35–50 minutes (30 minutes for completing the tasks and an optional amount of time for game-world exploration). An additional standardized external reward system—receiving game-related stickers, reward ribbons and medals for completing sessions (the same for all participants)—is used to even further raise the child’s motivation to do the training (for more details see [44] and S1 Appendix). In the current study BGB was presented in three conditions:

#### Full-active condition

**Fig 1:**
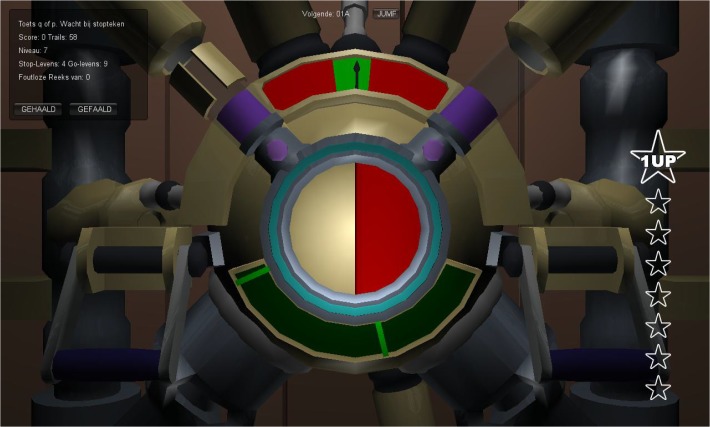
The inhibition training task with the green colored time-frame (response window) in the upper-middle of the screen.

In this condition WM, inhibition and cognitive-flexibility were all in training-mode. Training-mode entailed that, after each block of training tasks, the difficulty level of the training task was automatically adjusted to the child’s level of performance. Furthermore, in training-mode (a) the WM task [60] consisted of five training levels: the first level targeted visuospatial short-term memory (STM) only, whereas the other four levels targeted combinations of visuospatial STM, updating and manipulation of information (i.e. these four levels targeted both STM and the central executive). Each level was trained for 5 of the 25 sessions. The difficulty level was increased by increasing the amount of information that had to be remembered, updated and manipulated, (b) the inhibition task [61] was designed to decrease the time needed to inhibit a prepotent response (comparable with the stop signal reaction time measured by the STOP task [62]). On most trials the child had to respond to a go-stimulus by pressing left or right within a specific time-frame (a green colored response window between 550–850 ms; see Fig 1). This created a prepotent response tendency. However, on 25% of the trials, somewhere after the go-stimulus and before the middle of the response window, a stop-signal was presented (a tone and a visual cue) and the child had to inhibit the prepotent response (stop-trials). The difficulty level was increased by shortening the time allowed to inhibit this response, (c) the cognitive-flexibility task [61] was designed to decrease the time a child needs to adapt his/her behavior when task-rules change (i.e. switch cost). Specifically, the child had to sort objects with different shapes and colors (e.g. blue or red colored plungers and wheels) to either the left or the right according to a rule. The rule was either to sort according to shape or to sort according to color. In 25% of the trials the rule switched (switch-trials). The difficulty level was increased by shortening the time allowed to switch between the two rules (for a more detailed description of the three training tasks see [31]).

#### Partially-active condition

In this condition the inhibition and cognitive-flexibility tasks were in training-mode, and the WM task was in placebo-mode. Placebo-mode entailed that only the first level of the WM task was presented (for all 25 sessions), and that the difficulty level was not adjusted to the child’s level of performance (no more than two items had to be remembered). The amount of trials in placebo-mode was increased to match the training time in training-mode (10 minutes training per session for each EF domain).

#### Placebo condition

In this condition WM, inhibition and cognitive-flexibility were all in placebo-mode. In placebo-mode the inhibition task and the cognitive-flexibility task were presented the same way as in training-mode except that the stop-trials and switch-trials were replaced by go-trials and non-switch trials (i.e., no stop-trials and switch-trials were presented) and the difficulty level was not adjusted.

### Process Measures

No important changes to outcome measures were made after trial commencement.

#### Compliance

Compliance was defined as completing all of the 25 training sessions within a 5-week period. Using this algorithm, each child was categorized as compliant or noncompliant to treatment.

#### Blinding

At post-test, parents were asked to report the condition they thought their child was assigned to (full-active, partially-active, or placebo).

#### Improvement index during training

To validate whether the training actually improved task performance on the designated EFs, the improvement on training performance from beginning to end of training was assessed. It was tested whether children improved during training with paired t-tests. For the inhibition training and the cognitive flexibility training the results of day 2 and 3 of training (the Start Index) were compared with the results of their two best training days (the Max Index). The WM training had five levels and each level covered only 5 of the 25 training days. Therefore, to measure improvement on the WM training, within each level, the results of day 2 of training (the Start Index) were compared with the results of the best training day (the Max Index).

### Performance Measures

#### Stop task

The Stop task was used to measure the time needed to inhibit an ongoing response [62]. Two types of trials were presented: go-trials and stop-trials. During go-trials a go-stimulus (an arrow) that was either pointing right or left was presented. Participants were instructed to press a response button that corresponded to the direction of the stimulus as quickly and as accurately as possible. Stop-trials were identical to the go-trials but in addition a stop-signal was presented (a tone and a visual cue), which indicated that the participant had to withhold his/her ongoing response. The delay between the go- and stop-signal was dynamically varied (in steps of 50ms) so that inhibition was successful in 50% of the stop-trials. At this point, the go-process and stop-process are of equal duration, which makes it possible to estimate the latency of the stop-process: the stop signal reaction time (SSRT [62]). Aside from two practice blocks, four experimental blocks (of 64 trials each) were administered. The SSRT was used as outcome measure of inhibitory processing. Test retest reliability of the SSRT in children with ADHD is. 72 [63].

#### Stroop

The Stroop Color and Word Test [64] measures interference control and consists of three pages with words and/or colors. On the first page, word naming is measured by naming the words red, green, yellow, and blue, printed in black ink. On the second page, color naming is measured by naming the colors of small rectangles. The first and second page represent the congruent trials. On the third page, colors are then named when shown as nonmatching color words (incongruent trials). The interference score on the Stroop is the time needed for the third page minus the time needed for the second page, and was used as our outcome measure of interference control. The STROOP has adequate reliability [65].

#### Corsi Block Tapping Task (CBTT)

The CBTT [66] assesses the capacity of visuospatial STM and WM. The task consists of nine cubes (blocks) that are positioned on a board. In the present study, the same test format (size of board and blocks, distances between blocks) was used as in Kessels, van Zandvoort, Postma, Kappelle, and de Haan [67] (also see [68]), and the same procedure was used as in Geurts, Verté, Oosterlaan, Roeyers, and Sergeant [69]. The experimenter tapped a sequence of blocks that a child then had to reproduce in the same (CBTT-forward) or in reversed order (CBTT-backward). The minimum sequence length was three and the maximum was eight blocks, and each length was presented on three trials. The total amount of sequences that is correctly reproduced is the total score. The total score on the CBTT-forward (max. total score = 18) was used as an outcome measure for visuospatial STM and the total score on the CBTT-backward (max. total score = 18) was used as an outcome measure of visuospatial WM. The CBTT shows good reliability [70].

#### Digit span

The scaled score on the Digit-span subtest from the WISC-III testing battery [56] was used as a composite measure of verbal STM and WM. Participants were orally given sequences of numbers and were asked to repeat them, either in the same (i.e. STM) or in reversed order (i.e. WM). Digit span has adequate reliability [56].

#### Trail Making Test (TMT)

The TMT of the Delis-Kaplan Executive Function System (D-KEFS [71]) measures cognitive flexibility and is a timed task that requires the individual to connect a series of letters and numbers in ascending order while alternating between numbers and letters. The scaled contrast score—the contrast between the scaled non-switch trials (number- and letter sequencing) and the scaled switch trials (number-letter switching)—was used as outcome measure of cognitive flexibility (i.e., switch-cost). Test-retest reliabilities range from. 20 to. 77 [71].

#### Raven coloured progressive matrices

Raven’s coloured progressive matrices [72] measures non-verbal reasoning ability. The test consists of 36 items. The total amount of items correct (total score; max. = 36) was used as outcome measure for non-verbal reasoning. Test-retest reliability ranges from. 68 to. 90 [73].

### Questionnaires and Rating Scales

#### DBDRS (parent and teacher versions)

The DBDRS contains four DSM-IV scales; Inattention, Hyperactivity/ Impulsivity, Oppositional Defiant Disorder (ODD), and CD. Parents and teachers rate the child’s behavior on a 4-point Likert-type scale. Adequate psychometric properties have been reported [54]. The scores on the Inattention and Hyperactivity/Impulsivity scales were used as outcome measure of ADHD behavior. The scores on the ODD and CD scales were used as outcome measures of general problem behavior.

#### Behavior Rating Inventory of Executive Function questionnaire (BRIEF)

[74]. The Dutch version of the BRIEF is used to assess parent-rated EF. The BRIEF consists of 75 questions and includes eight EF sub-domains: Inhibit, Shift, Emotional Control, Initiate, WM, Plan/Organize, Organization of Materials, and Monitor. The test has adequate psychometric properties [75]. T-scores on the EF sub-domains were used as outcome measures.

#### Sensitivity to Punishment and Sensitivity to Reward Questionnaire for children (SPSRQ-C)

The SPSRQ-C measures parent-rated sensitivity to punishment and reward [76] (Dutch translation: [77]) and contains 33 items, divided in a Punishment Sensitivity scale, and three Reward Sensitivity scales: Reward Responsivity, Impulsivity/Fun-Seeking, and Drive. Each item is scored on a 5-point Likert scale. Adequate psychometric properties are reported [76]. Subscale scores were used as outcome measures.

#### Pediatric Quality of Life Inventory (PedsQL; parent and child versions)

[78] (Dutch translation: [79]). The PedsQL consists of 23 items, scored on a five-point Likert-scale, and is divided in four subscales: Physical, Emotional, Social, and School Functioning. The Psychosocial Health Summary score (a composite of the Emotional, Social and School Functioning subscales) was used as outcome measure. Adequate psychometric properties are reported [79].

#### The Home Situations Questionnaire (HSQ)

The HSQ [80] is designed to assess the impact of problem behavior at home and in public situations. Parents report whether each of 16 daily situations (e.g. getting dressed and going to bed) was a problem and rate their severity on a 9-point scale. The mean severity score was used as outcome measure. The HSQ has adequate psychometric properties [81].

### Procedure

This study was approved by the faculty’s IRB (the Ethics Review Board of the Faculty of Social and Behavioral Sciences of the university of Amsterdam). After obtaining written informed consent from the parents (on behalf of the participating children), parents and teachers completed the DBDRS. At this first screening the 6-month version of the DBDRS was administered (regarding the child’s behavior over the past 6-months), whereas at the pre-test, post-test and follow-up a two-week version of the DBDRS was administered (regarding the child’s behavior over the past two-weeks). If DBDRS inclusion criteria were met, children and parents were invited to the intake session. During this session questions regarding demographics were asked (see Table 1), and the PDISC-IV, and the short-form of the WISC-III were administered. The Chessboard WM task (for a detailed description see Dovis et al., 2013) was also administered during the intake session. However, this task was part of a different study and its results will therefore be reported elsewhere. If inclusion criteria were met, parent and child were invited to the pre-test session and the startup session, and were independently allocated to one of the three treatment conditions using the process of randomization by minimization [82] on the basis of age, gender, IQ, medication-use (yes/no), and parent- and teacher-rated inattention and hyperactivity/impulsivity symptoms (using the 6-months DBDRS). During the pre-test session the outcome measures were administered, and in the same week the teacher completed the two-week version of the DBDRS. The pre-test occurred approximately 1–2 weeks prior to the startup session (which was the start of the training). During the startup session parent and child were instructed about the training program, the computer, and the external reward system (see S1 Appendix), and a schedule for implementing the intervention and for weekly coaching calls was established. Once a research assistant completed a startup session with a particular family, he/she could not test or have further contact with that family or the teacher (to preserve blinding). During the 5-week, home-based training, a coach (a research assistant blind to the treatment condition) made weekly calls (of about 15 minutes; using a standardized telephone protocol) to the participating families to monitor progress, motivation and compliance, and to solve technical and game-related problems. Parents and children were explicitly instructed not to discuss the content of the training tasks with the coach. If a coach did receive information revealing the treatment condition, he/she was replaced and could no longer have contact with the family or the teacher. 1–2 weeks after the final training session the post-test was scheduled and the teacher completed the DBDRS. 3-months after the final training session the follow-up was scheduled and the teacher completed the DBDRS. At each test-session experimenters were blind to condition.

### Statistical Analyses

Sample size was determined by a prospective power analyses for univariate testing (using G*Power) based on the effect sizes of two previous EF-treatment studies [86], [45]. These studies suggested that the treatment effects on our primary outcome measures (i.e., EF measures, ADHD rating-scales) would be medium in size. Groups did not differ with respect to any of the baseline demographics or clinical characteristics (see Table 1). Also, including these baseline demographics and clinical characteristics (i.e., Gender, Age, FSIQ, DBDRS parent and teacher ratings, ODD diagnosis, ADHD medication use, Computergame experience, and Dyslexia) as covariates in the main analyses did not change the pattern of our results. Because repeated-measures were used, covariates were entered after mean centering (see [97]). Multinomial logistic regression was used for assessing the effectiveness of blinding.

An Intent-To-Treat (ITT) approach, using single imputations, was used to compare treatment effects of the three treatment conditions. That is, for each treatment group stochastic regression imputation was used to predict the missing posttest and follow-up values. The missing posttest values were based on the non-missing pretest and posttest scores of each treatment group. The missing follow-up values were based on the non-missing pretest scores, posttest scores, follow-up scores, and pretest-posttest difference scores of each treatment group (although the overall percentage of missing data was low—less than 5% was missing—it must be noted that stochastic regression imputation can increase the probability of making type I errors^).^

The dependent measures were subjected to four repeated measures MANOVAs (for the performance measures, for ADHD behavior, for EF and motivational behavior, and for general problem behavior; the covariance matrices were assumed to be unstructured), with Treatment condition (full-active, partially-active, placebo) as between-subject factor and Time (pre-test, post-test, follow-up) as within-subject factors. Bonferroni corrections for multiple testing were applied to these MANOVAs: only p-values < .0125 [.05/4] were considered significant. Trends and significant effects were further analyzed with simple contrasts. Bonferroni corrections for multiple testing were applied to these contrasts, in which the amount of dependent variables corrected for was defined per repeated measures MANOVA (7 performance-, 4 ADHD behavior-, 12 EF and motivational behavior-, and 7 general problem behavior variables were each analyzed in 3 pair-wise time comparisons [pre-test vs. post-test, post-test vs. follow-up, and pre-test vs. follow-up], resulting in a required significant level of *p* = .0024 [.05/21] for the performance measures contrasts, *p* = .0042 [.05/12] for the ADHD behavior contrasts, *p* = .0014 [.05/36] for the EF and motivational behavior contrasts, and *p* = .0024 [.05/21] for the general problem behavior contrasts). For additional within-group analyses paired t-tests were used (Bonferroni corrections were applied). Partial Eta squared effect sizes (*η*_*p*_^2^) are reported for all analyses: *η*_*p*_^2^ = .01 is regarded a small effect size,. 06 a medium effect size, and. 14 a large effect size [83].

## Results

### Process Measures

#### Compliance during training

**Fig 2:**
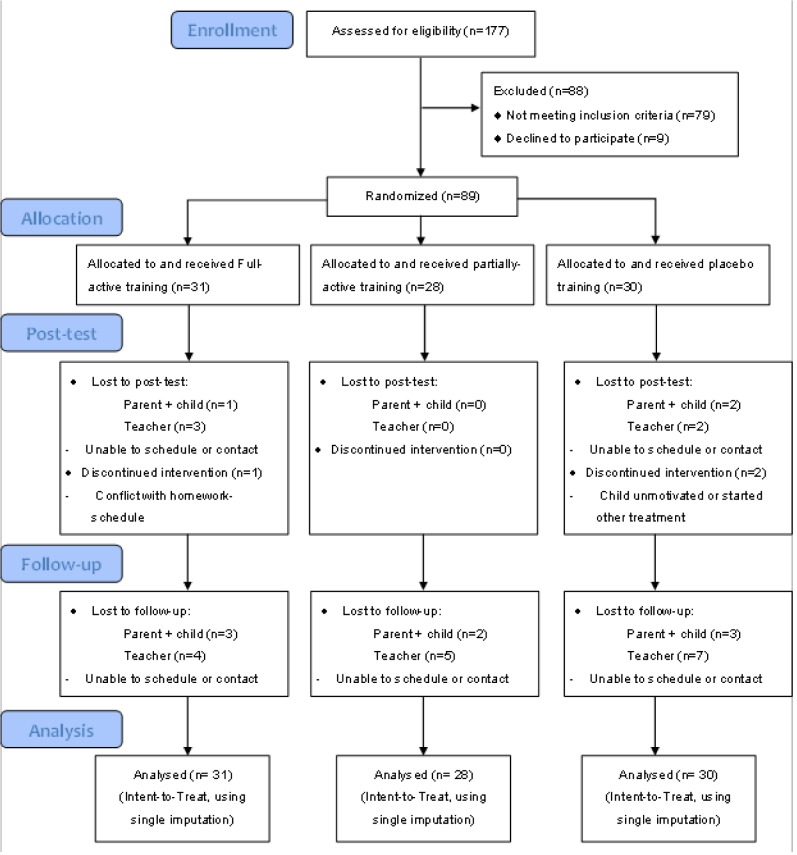
CONSORT flow diagram.

Of the 31 participants assigned to the full-active condition, 30 (96.7%) met compliance criteria (25 training days within 5 weeks). All of the 28 participants assigned to the partially-active condition met compliance criteria. Of the 30 participants assigned to the placebo condition, 28 (93.3%) met compliance criteria. Overall, compliance to treatment was high, given that this was a home-based intervention that included a substantial portion of participants with ODD (see Fig 2).

#### Post-training dropout

Eight participants (9%) of our total sample (i.e., 3 children in the full-active condition, 2 children in the partially-active condition, and 3 children in the placebo condition) were lost to post-test and follow-up testing (see Fig 2). There were no significant differences on baseline demographics and clinical characteristics (i.e., gender, age, IQ, DBDRS parent and teacher ratings, ODD-diagnosis, medication use, computergame experience, Dyscalculia and Dyslexia) between the children lost to post-test and follow-up testing and the children who participated in these assessments (depending on the level of measurement a MANOVA or Pearson’s chi-squared tests were used). But note that the sample size of the post-training drop out group was small.

#### Blinding

There was no significant association between the conditions wherein participants were actually included and the conditions whereof parents afterwards reported (guessed) that their child was assigned to (the multinomial logistic regression model indicated a non-significant model overall, χ^2^(4) = 1.26, *p* = .868, -2LL = 18.004). This suggests that, based upon their experience with the actual training condition, parents were not able to guess the condition wherein their child was included. Further, no participant (child, parent, teacher, experimenter, or coach) was unblinded at any point during the conduct of the trial.

#### Improvement index during training

It was tested whether children improved during training with paired t-tests (Bonferroni corrections for multiple testing were applied: only p-values < .0024 [.05/21] were considered significant). Within the full-active condition, paired t-tests showed a significant difference (improvement) between the Start Index and Max Index for the Inhibition training, t (30) = -18.66, *p* < .001, the Cognitive flexibility training, t (30) = -19.14, *p* < .001, and for all the levels of the WM training (level 1, t (30) = -7.25, *p* < .001; level 2, t (30) = -7.90, *p* < .001; level 3, t (30) = -7.19, *p* < .001, level 4, t (30) = -9.21, *p* < .001; level 5, t (30) = -7.72, *p* < .001). Within the partially-active condition (where WM was in placebo-mode), paired t-tests showed significant difference (improvement) between the Start Index and the Max Index for both the Inhibition training, t (27) = -15.86, *p* < .001, and the Cognitive flexibility training, t (27) = -22.89, *p* < .001.

### Performance Measures

A 3x3 (Treatment condition x Time [pre-test, post-test, follow-up]) repeated measures MANOVA with the main scores of the EF tasks (Stoptask [SSRT], STROOP [interference score], CBTT-fwd [total score], CBTT-bkw [total score], Digit recall [scaled score], TMT [scaled contrast score]) and the Raven (total score) as dependent variables (scores on all seven tasks were analyzed simultaneously), showed a main effect of Time, *F* (14,334) = 6.74, *p* < .001, *η*_*p*_^2^ = .22, no main effect of Treatment condition, *F* (14,162) = 1.41, *p* = .154, *η*_*p*_^2^ = .11, and a non-significant trend towards an interaction between Treatment condition and Time, *F* (28,676) = 1.59, *p* = .027, *η*_*p*_^2^ = .06 (after Bonferroni correction only p-values < .0125 were considered significant). To interpret these effects for each performance based measure, we used simple contrasts:

For each performance based measure, main Time effects and Treatment condition x Time interactions are presented per pair-wise time difference (i.e. pre- vs. post-test, post- vs. follow-up test; pre- vs. follow-up test) in Table 2.

**Table 2:** Outcomes at Baseline, Post-test and Follow-up.

	Full-active Condition						Partially-active Condition						Placebo Condition						*Time constrasts. F*(1. 86)						*Treatment* *Time constrasts. F(2. 86)					
	Pre		Post		FU		Pre		Post		FU		Pre		Post		FU		Pre vs. Post		Post vs. FU		Pre vs. FU		Pre vs. Post		Post vs. FU		Pre vs. FU	
Domain and Measure	*M*	*SD*	*M*	*SD*	*M*	*SD*	*M*	*SD*	*M*	*SD*	*M*	*SD*	*M*	*SD*	*M*	*SD*	*M*	*SD*	*F*	η_*p*_^*2*^	*F*	η_*p*_^*2*^	F	η_*p*_^*2*^	F	η_*p*_^*2*^	F	η_*p*_^*2*^	F	η_*p*_^*2*^
**Performance Measures**																														
Stoptask (SSRT)	189.6	43.7	151.4	39.7	147.4	40.9	197.0	65.2	158.8	31.6	150.2	48.7	200.1	73.8	197.7	69.0	189.0	68.6	16.50***	.16	1.18	.01	18.41***	.18	3.42*	.07	.06	.001	2.08	.05
STROOP (interfence score)	68.8	33.9	53.2	24.1	46.2	19.9	70.5	33.0	49.4	23.7	53.2	25.3	68.0	30.4	55.9	26.4	62.6	33.0	28.50***	.25	.17	.002	23.27***	.21	.71	.02	2.29	.05	2.69†	.06
CBTT-forward (total score)	9.6	2.4	11.7	2.3	11.0	2.4	9.5	2.2	10.1	2.3	9.7	2.5	9.9	2.9	10.1	1.9	9.6	2.1	18.08***	.17	10.13**	.11	2.72	.10	6.96**	.14	.21	.01	4.59*	.10
CBTT-backward (total score)	8.7	2.3	10.1	2.9	10.0	2.3	9.1	2.5	9.4	2.0	9.3	2.3	9.2	2.5	9.2	2.3	9.4	2.3	5.33*	.06	.004	<.001	4.87*	.05	3.36*	.07	.23	.01	2.53	.06
Digit Recall (scaled score)	9.3	2.9	9.9	3.3	9.9	3.1	9.1	3.2	9.9	3.3	9.6	3.3	9.8	2.6	9.9	2.9	9.5	3.1	3.12	.04	.45	.01	.98	.01	.47	.01	.21	.01	.95	.02
TMT (scaled contrast score)	8.9	3.1	8.6	2.8	8.6	2.8	8.7	3.1	8.9	3.5	9.0	2.3	8.7	2.6	8.6	2.4	8.7	2.1	.02	<.001	.002	<.001	.01	<.001	.16	<.001	.02	<.001	.23	.01
Raven (total score)	33.0	2.7	33.5	2.1	34.2	1.7	32.1	3.5	32.9	3.3	33.6	2.5	33.2	2.2	33.7	2.0	33.7	1.9	6.86*	.07	5.21*	.06	25.81***	.23	.11	.002	1.50	.03	2.04	.05
**ADHD Behavior**																														
P-DBDRS Inattention	17.5	4.3	11.9	5.7	12.9	4.1	17.7	5.5	12.6	4.5	14.6	5.3	18.2	4.4	13.6	5.2	14.1	4.7	66.21***	.44	5.31*	.06	48.37***	.36	.23	.01	.92	.02	.69	.02
P-DBDRS Hyp/Imp	17.0	5.3	12.2	6.6	12.6	6.4	16.5	4.5	12.0	5.3	13.0	5.1	17.0	4.7	12.9	6.5	12.5	5.7	63.01***	.42	.55	.01	55.70***	.39	.11	.003	.85	.02	.37	.01
T-DBDRS Inattention	14.0	4.5	11.7	5.6	12.2	5.8	14.7	5.0	11.0	5.6	13.3	6.6	13.8	5.4	12.3	5.0	11.3	5.1	19.27***	.18	.89	.01	10.11**	.11	1.20	.03	2.20	.05	.30	.01
T-DBDRS Hyp/Imp	12.7	6.4	11.1	5.5	9.3	4.9	13.1	6.6	10.0	6.2	11.5	7.0	12.4	5.1	11.6	6.0	9.1	4.0	13.29***	.13	2.65	.03	20.94***	.20	1.66	.04	4.77*	.10	.93	.02
**EF- & Motiv. Behavior**																														
P-BRIEF Inhibit	70.5	11.5	63.8	10.9	63.1	10.9	69.8	7.6	65.6	10.1	65.8	10.4	71.5	9.4	63.8	10.2	62.7	10.4	37.42***	.30	.51	.01	41.24***	.32	1.05	.02	.25	.01	1.85	.04
P-BRIEF Working Memory	68.0	6.8	59.1	9.6	60.9	8.9	67.1	7.7	60.3	9.3	62.4	8.3	67.8	6.8	59.5	6.6	61.1	8.1	94.93***	.53	5.82*	.06	81.63***	.49	.61	.01	.04	.001	1.21	.03
P-BRIEF Shift	58.8	10.5	53.1	8.1	51.8	10.2	58.8	11.1	54.3	11.8	53.1	10.1	64.6	9.6	56.7	8.2	54.8	8.5	40.34***	.32	4.08*	.05	60.10***	.41	1.09	.03	.09	.002	1.55	.04
P-BRIEF Emotional Control	59.4	11.0	53.1	10.5	54.0	11.0	59.2	11.1	56.7	12.9	56.5	12.2	63.4	9.3	57.2	10.6	55.7	11.4	27.45***	.24	.07	.001	25.84***	.23	1.71	.04	.56	.01	1.97	.04
P-BRIEF Initiate	58.9	9.2	53.3	10.1	53.3	10.5	58.3	6.2	53.1	9.8	54.4	8.2	62.4	9.0	54.7	9.3	56.3	10.8	45.23***	.35	1.05	.012	38.56***	.31	.73	.02	.26	.01	.66	.02
P-BRIEF Plan/Organize	61.5	8.7	56.4	9.0	55.9	8.2	61.6	7.7	56.8	9.2	57.8	9.5	63.1	7.3	58.1	7.6	59.4	7.0	32.26***	.27	.54	.01	29.66***	.26	.01	<.001	.44	.01	.56	.01
P-BRIEF Organiz. Materials	54.5	10.0	51.8	12.4	52.0	11.1	58.5	6.2	56.5	8.2	55.1	10.4	55.8	9.5	52.6	9.9	54.5	9.8	9.87**	.10	.08	.001	6.23*	.07	.18	.004	1.19	.02	.42	.01
P-BRIEF Monitor	63.5	5.6	58.2	7.1	60.6	8.3	63.1	7.5	59.4	10.3	59.5	9.3	65.4	5.5	58.5	7.9	60.3	8.2	34.30***	.29	2.83	.03	19.72***	.19	.99	.02	.64	.02	.58	.01
P-SPSRQ Punish. Sens.	2.6	0.6	2.4	0.5	2.4	0.6	2.3	0.5	2.2	0.5	2.4	0.6	2.8	0.6	2.6	0.9	2.6	0.7	5.46*	.06	1.33	.02	2.08	.02	.14	.003	.73	.02	1.52	.03
P-SPSRQ Imp/Fun Seeking	3.2	0.6	3.0	0.5	3.0	0.4	3.3	0.5	3.1	0.5	3.3	0.6	3.4	0.6	3.2	0.6	3.3	0.7	10.95**	.11	3.39†	.04	3.19	.04	.28	.01	1.21	.03	.36	.01
P-SPSRQ Reward Respons.	3.7	0.6	3.6	0.6	3.4	0.6	3.8	0.6	3.6	0.7	3.7	0.6	3.6	0.7	3.6	0.8	3.6	0.7	3.78†	.04	.09	.001	7.65**	.08	.66	.02	2.06	.05	1.01	.02
P-SPSRQ Drive	3.1	0.9	3.2	0.9	3.0	0.8	3.5	0.8	3.5	0.7	3.6	1.0	3.4	0.9	3.1	1.1	3.2	0.8	1.06	.01	.17	.002	1.94	.02	2.45	.06	1.30	.03	1.45	.03
**Gen. Problem Behavior**																														
P-DBDRS ODD	8.3	4.8	6.0	5.1	7.0	5.3	10.0	5.1	8.3	5.3	8.6	4.6	9.4	4.2	6.9	4.1	6.9	4.7	25.35***	.23	1.93	.02	20.90***	.20	.25	.01	.65	.02	1.02	.02
P-DBDRS CD	1.1	1.6	0.7	1.0	1.0	1.7	1.5	1.5	0.8	1.3	1.3	1.4	1.6	1.9	1.2	1.6	0.8	1.5	13.29***	.13	1.44	.02	5.85*	.06	.45	.01	2.63	.06	1.72	.04
T-DBDRS ODD	6.6	5.0	5.9	4.9	5.3	4.6	6.0	4.7	4.5	4.3	5.8	5.7	6.8	6.0	5.1	5.4	4.3	4.7	10.02**	.10	.01	<.001	7.44**	.08	.66	.02	1.89	.04	1.68	.04
T-DBDRS CD	1.2	1.6	1.1	1.9	1.5	2.4	1.6	2.3	1.5	2.4	1.2	2.1	1.9	2.6	1.1	1.8	1.0	1.6	3.55†	.04	.01	.001	2.73	.03	1.96	.04	.89	.02	2.70	.06
P-PEDsQL Psy.soc. Hlth.	61.8	12.1	73.1	13.9	72.6	9.1	61.0	14.1	69.0	14.3	65.3	12.9	51.3	14.5	63.8	14.9	62.2	15.6	53.48***	.38	1.84	.02	47.39***	.36	.81	.02	.87	.01	2.77†	.06
C-PEDsQL Psy.soc. Hlth.	67.2	17.3	67.8	13.5	66.9	15.1	68.3	14.1	70.0	16.2	70.7	13.5	63.7	11.7	67.2	15.3	66.3	15.4	2.88	.03	.18	.001	2.06	.02	.54	.01	.18	.004	.71	.02
P-HSQ Mean Severity Score	4.3	1.8	3.6	1.8	3.8	1.9	4.2	1.8	3.5	1.6	3.7	2.0	4.7	1.5	3.7	1.5	3.5	1.5	15.71***	.15	.12	.001	12.69**	.13	.28	.01	.55	.02	1.36	.03

*Note*. BRIEF = Behavior Rating Inventory of Executive Function; C- = Child-rated; CBTT = Corsi Block Tapping Task; CD = conduct disorder; DBDRS = Disruptive Behavior Disorder Rating Scale; FU = Follow-up-test (after 3 months); HSQ = Home Situations Questionnaire; Imp/Fun Seeking = Impulisivity/Fun Seeking; ODD = oppositional defiant disorder; Organiz. Materials = Organization of Materials; P- = Parent-rated; PEDsQL = Pediatric Quality of Life Inventory; Post = Post-test; Pre = Pre-test; Psy.soc. Hlth. = Psychosocial Health Summary Score; Punish. Sens. = Punishment Sensitivity; Reward Respons. = Reward Responsiveness; SPSRQ = Sensitivity to Punishment and Sensitivity to Reward Questionnaire for children; SSRT = Stop Signal Reaction Time; T- = Teacher-rated; TMT = Trail Making Task;

* *p* < .05;

** *p* < .01;

*** *p* < .001;

† *p* < .075.

After Bonferroni correction (*p* < .0024 [.05/21]) results indicate the following: Between the pre- and post-test there was a significant Treatment condition x Time interaction for the CBTT-fwd (*p* = .002), and a non-significant trend for the Stoptask (*p* = .037) and the CBTT-bkw (*p* = .039; see Table 2). Between the pre-test and follow-up there was a non-significant trend towards a Treatment condition x Time interaction for the CBTT-fwd (*p* = .013) and the STROOP (*p* = .07; see Table 2). Other pair-wise time differences in Treatment condition x Time interaction effects were non-significant both with- and without Bonferroni correction (investigating Digit recall forward and backward separately [using raw scores] did not change the results). Next, in order to obtain more insight into these two-way interactions, three follow-up repeated measures MANOVAs were performed: one for each combination of treatment conditions (Bonferroni corrections were applied: only p-values < .0167 [.05/3] were considered significant).

#### Full-active condition versus placebo condition

**Fig 3:**
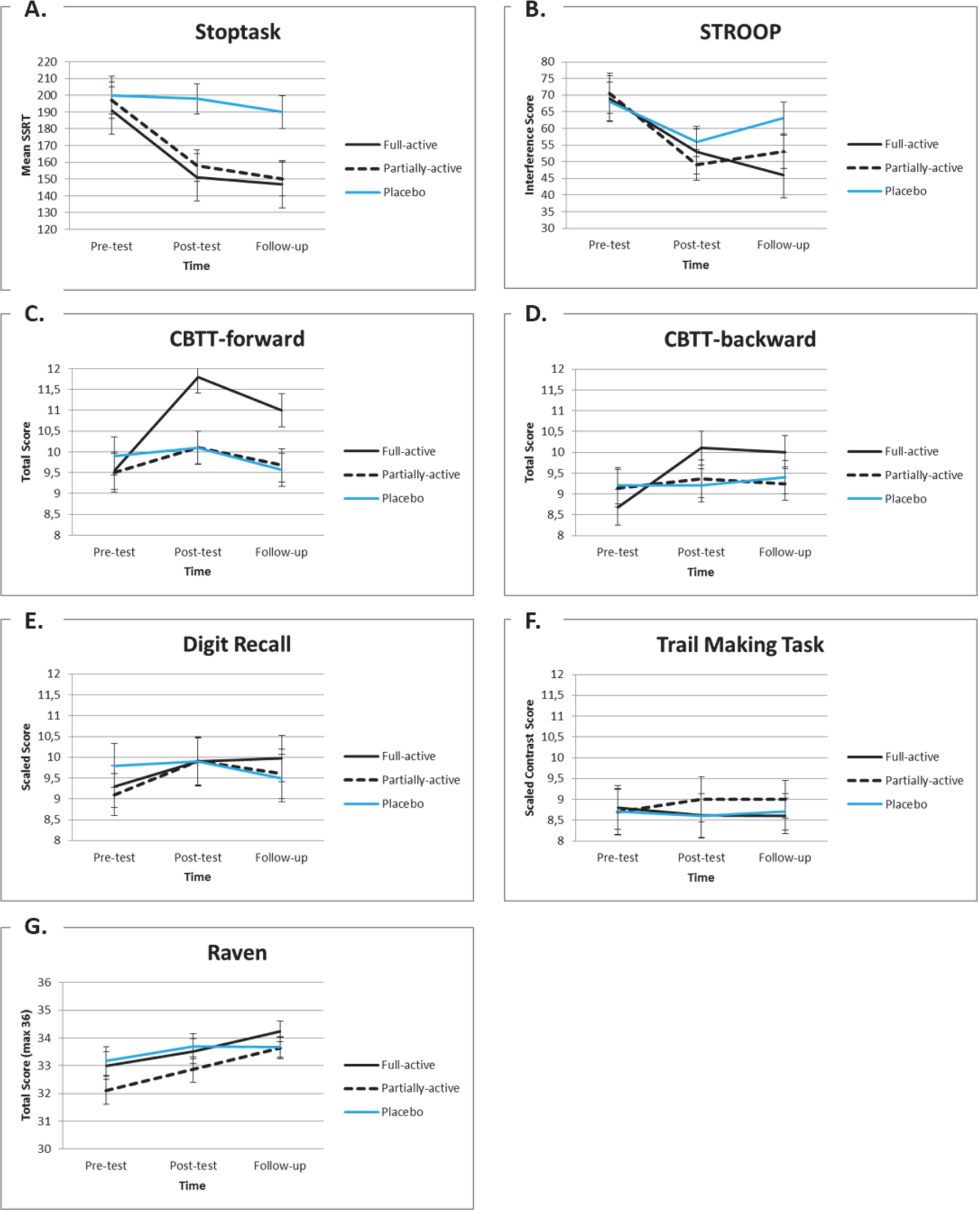
Mean values and standard errors of the executive functioning tasks (A—F) and the Raven (G) on the pre-test, post-test and (3 month) follow-up of children in the three treatment groups. *Note*: SSRT = Stop Signal Reaction Time; CBTT = Corsi Block Tapping Task.

A 2x3 (Treatment condition x Time) repeated measures MANOVA with the main scores of the Stoptask, STROOP, CBTT-fwd, and CBTT-bkw as dependent variables, showed a main effect of Time, *F* (8,232) = 6.22, *p* < .001, *η*_*p*_^2^ = .18, a main effect of Treatment condition, *F* (4,56) = 5.06, *p* = .009, *η*_*p*_^2^ = .21, and a significant interaction between Treatment condition and Time, *F* (8,232) = 3.90, *p* < .001, *η*_*p*_^2^ = .12. To further interpret this interaction for each relevant pair-wise time difference and each performance based measure, we used simple contrasts (in the previous contrast analyses no Treatment x Time interactions were found between post-test and follow-up; therefore, only the two-way interactions between pre-test and post-test and between pre-test and follow-up were further explored):

These contrasts are presented in Table 3 and indicate that, compared to pre-test performance, post-test- and/or follow-up performance on the Stoptask, the STROOP and the CBTT forward and backward improved more in the full-active condition than in the placebo condition (*p*-values ranged from. 002 to. 020; effect sizes ranged from medium to large; see Table 3 and Fig 3A–3D). However, after Bonferroni correction only the Treatment x Time interactions for the CBTT-fwd remained significant (as only p-values < .0063 [.05/8] were considered significant).

**Table 3:** Outcome of repeated measures MANOVAs contrasts for task performance in each combination of treatment conditions.

	Full-active vs. Placebo				Partially-active vs. Placebo				Full-active vs. Partially-active			
	Treatment*Time constrasts, *F*(1,59)				Treatment*Time constrasts, *F*(1,56)				Treatment*Time constrasts, *F*(1,57)			
	Pre vs. Post		Pre vs. FU		Pre vs. Post		Pre vs. FU		Pre vs. Post		Pre vs. FU	
Measure	*F*	*η*_*p*_^*2*^	*F*	*η*_*p*_^*2*^	*F*	*η*_*p*_^*2*^	*F*	*η*_*p*_^*2*^	*F*	*η*_*p*_^*2*^	*F*	*η*_*p*_^*2*^
Stoptask (SSRT)	5.73*	.09	2.63	.04	4.22*	.07	2.68	.05	<.001	<.001	.09	.002
STROOP (interference)	.25	.004	6.53*	.10	1.16	.02	2.26	.04	.60	.01	.39	.01
CBTT-forward	11.03**	.16	8.35**	.12	.83	.02	.77	.01	6.92*	.11	4.15*	.07
CBTT-backward	5.98*	.09	2.91	.05	.19	.003	.02	<.001	3.71†	.06	5.76*	.09

*Note*. CBTT = Corsi Block Tapping Task; FU = Follow-up-test (after 3 months); Post = Post-test; Pre = Pre-test; SSRT = Stop Signal Reaction Time;

* *p* < .05;

** *p* < .01;

† *p* < .06.

### Partially-active condition versus placebo condition

A 2x3 (Treatment condition x Time) repeated measures MANOVA with the main scores of the Stoptask, STROOP, CBTT-fwd, and CBTT-bkw as dependent variables, showed a main effect of Time, *F* (8,220) = 3.49, *p* = .001, *η*_*p*_^2^ = .11, no main effect of Treatment condition, *F* (4,53) = 1.44, *p* = .235, *η*_*p*_^2^ = .10, and no significant interaction between Treatment condition and Time, *F* (8,220) = 1.07, *p* = .388, *η*_*p*_^2^ = .04. However, since we had specific expectations regarding the Treatment condition x Time interactions—we only expected this interaction for the Stoptask and the STROOP, not for the CBTT forward and backward (as WM was not trained in either condition)—simple contrasts were used to further explore the non-significant interaction effect:

These contrasts are presented in Table 3 and indicate that, compared to pre-test performance, post-test performance on the Stoptask improved more in the partially-active condition than in the placebo condition (*p* = .045; medium effect size; see Table 3 and Fig 3A). However, this difference was no longer significant after Bonferroni correction: as only p-values < .0063 (.05/8) were considered significant.

### Full-active condition versus partially-active condition

A 2x3 (Treatment condition x Time) repeated measures MANOVA with the main scores of the Stoptask, STROOP, CBTT-fwd, and CBTT-bkw as dependent variables, showed a main effect of Time, *F* (8,224) = 9.79, *p* < .001, *η*_*p*_^2^ = .26, no main effect of Treatment condition, *F* (4,54) = 1.76, *p* = .151, *η*_*p*_^2^ = .12, and a non-significant trend towards an interaction between Treatment condition and Time, *F* (8,224) = 2.00, *p* = .048, *η*_*p*_^2^ = .07 (after Bonferroni correction only p-values < .0167 [.05/3] were considered significant). To further interpret this interaction, we used simple contrasts:

These contrasts are presented in Table 3 and indicate that, compared to pre-test performance, post-test and/or follow-up performance on the CBTT (forward and backward) improved more in the full-active condition than in the partially-active condition (*p*-values ranged from. 011 to. 046, effect sizes were medium; see Table 3 and Fig 3C and 3D). However, these differences were no longer significant after Bonferroni correction: as only p-values < .0063 (.05/8) were considered significant.

#### Within-group analyses

For each EF task where a Treatment condition x Time interaction was significant with or without Bonferroni correction (Stoptask, STROOP, CBTT-fwd, CBTT-bkw), differences within each treatment group between the pre- and post-test and between the pre-test and follow-up were tested with additional paired t-tests (Bonferroni corrections were applied: only p-values < .0021 [.05/24] were considered significant).

Results are presented in Table 4. After Bonferroni correction we found that: in the full-active condition performance on the Stoptask, the STROOP, the CBTT-fwd and the CBTT-bkw significantly improved between pre- and post-test. Performance on the Stoptask, the STROOP and the CBTT-bkw also significantly improved between pre-test and follow-up (there was a trend for performance on the CBTT-fwd, *p* = .003). In the partially-active condition performance on the STROOP significantly improved between pre- and post-test (there was a trend for performance on the Stoptask, *p* = .005), and performance on the Stoptask significantly improved between pre-test and follow-up (there was a trend for performance on the STROOP, *p* = .016). In the placebo condition none of the differences were significant (although there was a trend for STROOP performance between pre- and post-test, *p* = .043; see Table 4).

**Table 4:** Within-group comparisons of pair-wise time differences in task performance (using paired t-tests).

	Full-active		Partially-active		Placebo	
	Paired t-tests, t (30)		Paired t-tests, t (27)		Paired t-tests, t (29)	
	Pre vs Post	Pre vs FU	Pre vs Post	Pre vs FU	Pre vs Post	Pre vs FU
Measure	t	t	t	t	t	t
Stoptask (SSRT)	4.29***	4.64***	3.03**	3.53**	.20	.65
STROOP (interference)	3.91***	4.47***	3.49**	2.57*	2.12*	1.22
CBTT-forward	-4.70***	-3.25**	-1.88	-.41	-.43	.83
CBTT-backward	-3.39**	-3.49**	-.59	-.34	<.001	-.38

*Note*. CBTT = Corsi Block Tapping Task; FU = Follow-up-test (after 3 months); Post = Post-test; Pre = Pre-test; SSRT = Stop Signal Reaction Time;

* *p* < .05;

** *p* < .01;

*** *p* < .001.

### Questionnaires and Rating Scales

#### ADHD behavior (parent and teacher DBDRS)

**Fig 4:**
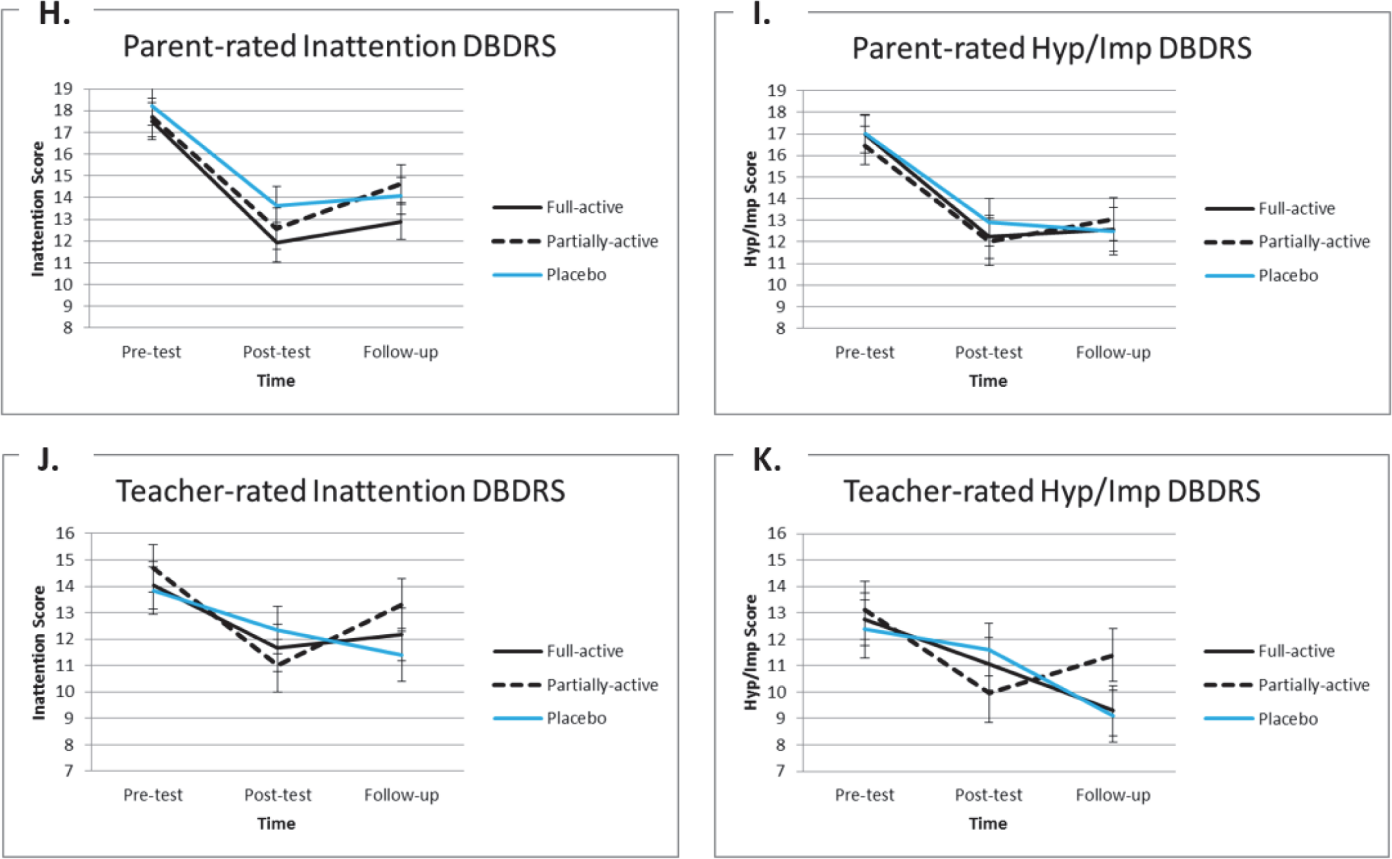
Mean values and standard errors of the mean scores on the Inattention and Hyperactivity/Impulsivity (Hyp/Imp) scales of the parent and the teacher versions of the Disruptive Behavior Disorder Rating Scale (DBDRS; H-K) on the pre-test, post-test and (3 months) follow-up of children in the three treatment groups.

A 3x3 (Treatment condition x Time) repeated measures MANOVA with mean scores on the Inattention and Hyperactivity/Impulsivity scales of the parent and the teacher version of the DBDRS as dependent variables, showed a main effect of Time, *F* (8,340) = 13.32, *p* < .001, *η*_*p*_^2^ = .24, no main effect of Treatment condition, *F* (8,166) = .33, *p* = .953, *η*_*p*_^2^ = .02, and no significant interaction between Treatment condition and Time, *F* (16,688) = .77, *p* = .718, *η*_*p*_^2^ = .02. The significant Time effect was further explored using simple contrasts:

For each ADHD scale, main Time effects are presented per pair-wise time difference in Table 2. After Bonferroni correction (*p* < .0042 [.05/12]) results indicate that: compared to the pre-test, both parents and teachers reported a significant decrease in ADHD symptoms at the post-test and at the follow-up (effect sizes of parent-ratings were large; effect sizes of teacher-ratings ranged from medium to large). However, the non-significant Treatment x Time interaction indicates that this decrease did not differ between the Treatment conditions (in addition see Table 2 & Fig 4).

#### Parent-rated EF- and motivational behavior (BRIEF and SPSRQ-C)

A 3x3 (Treatment condition x Time) repeated measures MANOVA with mean t-scores on the Inhibit-, Working Memory-, Shift-, Emotional Control-, Initiate-, Plan/Organize-, Organization of Materials-, and Monitor scales of the BRIEF, and the mean scores on the Sensitivity to Punishment-, Impulsivity/Fun Seeking-, Reward Responsiveness-, and Drive scales of the SPSRQ-C as dependent variables, showed a main effect of Time, *F* (30,318) = 4.91, *p* < .001, *η*_*p*_^2^ = .32, no main effect of Treatment condition, *F* (30,146) = 1.08, *p* = .368, *η*_*p*_^2^ = .18, and no significant interaction between Treatment condition and Time, *F* (60,644) = .72, *p* = .942, *η*_*p*_^2^ = .06. The significant Time effect was further explored using simple contrasts:

These contrasts are presented in Table 2. After Bonferroni correction (*p* < .0014 [.05/36]) results indicate the following: after training, parents reported a significant improvement (with large effect sizes) on almost all scales of the BRIEF (EF behavior; only improvement on the Organization of Materials scale was no longer significant after Bonferroni correction) and on the Impulsivity/Fun Seeking scale of the SPSRQ-C (motivational behavior; medium effect size; improvement on the Punishment Sensitivity scale [*p* = .022] and the Reward Responsiveness scale [*p* = .007] was no longer significant after Bonferroni correction). However, the non-significant Treatment x Time interaction indicates that these improvements did not differ between the Treatment conditions (in addition see the Treatment x Time contrasts in Table 2).

#### General problem behavior (DBDRS, PEDsQL, and HSQ)

A 3x3 (Treatment condition x Time) repeated measures MANOVA with mean scores on the ODD and the CD scales of the parent and the teacher version of the DBDRS, the Psychosocial Health Summary score of the parent and the child version of the PEDsQL, and the mean severity score of the parent-rated HSQ as dependent variables, showed a main effect of Time, *F* (14,334) = 5.15, *p* < .001, *η*_*p*_^2^ = .18, a non-significant trend towards a main effect of Treatment condition, *F* (14,162) = 1.83, *p* = .038, *η*_*p*_^2^ = .14 (after Bonferroni correction only p-values < .0125 were considered significant), and no significant interaction between Treatment condition and Time, *F* (28,676) = 1.10, *p* = .337, *η*_*p*_^2^ = .04. The significant Time effect was further explored using simple contrasts:

These contrasts are presented in Table 2. After Bonferroni correction (*p* < .0024 [.05/21]) results indicate the following: after training, parents reported a significant improvement on all general problem behavior indices (effect sizes ranged from medium to large), and teachers reported a significant improvement on the ODD scale of the DBDRS (medium effect size). However, the non-significant Treatment x Time interaction indicates that these improvements did not differ between the Treatment conditions (in addition see the Treatment x Time contrasts in Table 2). In contrast to their parents, children reported no significant difference in their Psychosocial Health Summary Score after training.

### Treatment Responders

In addition to the overall means, the percentage of children who benefitted from training was calculated for each measure that showed significant (with or without Bonferroni correction) main Time effects and/or Treatment condition x Time interactions on the pairwise comparisons of pre- and post-test scores and/or pre- and follow-up test scores (see Table 2). On each of these measures children were either classified as responders or non-responders by using reliable change indices [98], [99]. Based on classification guidelines by Wise [99], a participant was classified as responder when both the following criteria were met: (1) a reliable change index (RCI) of at least 1.28 (RCI was based on the method of [98]), and (2) an improvement of scores of at least 1 standard deviation [99]. Results for each treatment condition are presented in Table 5. The pattern of these results strongly resembles the pattern of the mean results (see Table 5; in addition see Table A in S2 Appendix).

**Table 5:** Proportion of treatment groups showing improvement on performance measures and rating-scales (i.e., responders).

	Full-active Condition		Partially-active Condition		Placebo Condition	
	Pre vs. Post	Pre vs. FU	Pre vs. Post	Pre vs. FU	Pre vs. Post	Pre vs. FU
Domain and Measure	*% responders*	*% responders*	*% responders*	*% responders*	*% responders*	*% responders*
**Performance Measures**						
Stoptask (SSRT)	***41*.*9***	**54.8**	***35*.*7***	***35*.*7***	16.7	20.0
STROOP (interfence score)	9.7	22.6	21.4	10.7	14.6	6.7
CBTT-forward (total score)	***48*.*4***	***41*.*9***	17.9	17.9	13.3	13.3
CBTT-backward (total score)	***38*.*7***	29.0	17.9	3.6	10.0	16.7
Raven (total score)	12.9	19.4	10.7	14.3	16.7	16.7
**ADHD Behavior**						
P-DBDRS Inattention	**51.6**	***48*.*4***	**53.6**	***32*.*1***	***50*.*0***	***50*.*0***
P-DBDRS Hyp/Imp	***45*.*2***	***38*.*7***	***50*.*0***	***42*.*9***	***40*.*0***	***46*.*7***
T-DBDRS Inattention	29.0	***32*.*2***	***42*.*9***	28.6	23.3	30.0
T-DBDRS Hyp/Imp	16.1	***38*.*7***	25.0	25.0	20.0	30.0
**EF- & Motiv. Behavior**						
P-BRIEF Inhibit	***35*.*5***	***35*.*5***	25.0	25.0	26.7	***43*.*3***
P-BRIEF Working Memory	**54.8**	***41*.*9***	***39*.*3***	***32*.*1***	**56.7**	***50*.*0***
P-BRIEF Shift	25.8	***32*.*3***	17.9	25.0	***40*.*0***	***50*.*0***
P-BRIEF Emotional Control	***32*.*3***	***32*.*3***	14.3	21.4	***40*.*0***	***36*.*7***
P-BRIEF Initiate	***35*.*5***	***38*.*7***	***50*.*0***	***39*.*3***	***43*.*3***	30.0
P-BRIEF Plan/Organize	29.0	29.0	28.6	***35*.*7***	***33*.*3***	***33*.*3***
P-BRIEF Organiz. Materials	22.6	19.4	25.0	28.6	23.3	16.7
P-BRIEF Monitor	***48*.*4***	***48*.*4***	25.0	***32*.*1***	**53.3**	***46*.*7***
P-SPSRQ Punish. Sens.	12.9	9.7	3.6	3.6	23.3	13.3
P-SPSRQ Imp/Fun Seeking	22.6	16.1	25.0	14.3	10.0	13.3
P-SPSRQ Reward Respons.	16.1	22.6	21.4	10.7	10.0	3.3
**Gen. Problem Behavior**						
P-DBDRS ODD	***32*.*3***	19.4	10.7	10.7	30.0	20.0
P-DBDRS CD	6.5	3.2	25.0	14.3	10.0	13.3
T-DBDRS ODD	6.5	***32*.*3***	21.4	17.9	16.7	20.0
P-PEDsQL Psy.soc. Hlth.	**51.6**	***48*.*4***	25.0	21.4	***40*.*0***	***36*.*7***
P-HSQ Mean Severity Score	22.6	25.8	14.3	21.4	30.0	30.0

*Note*. BRIEF = Behavior Rating Inventory of Executive Function; CBTT = Corsi Block Tapping Task; CD = conduct disorder; DBDRS = Disruptive Behavior Disorder Rating Scale; FU = Follow-up-test (after 3 months); HSQ = Home Situations Questionnaire; Imp/Fun Seeking = Impulisivity/Fun Seeking; ODD = oppositional defiant disorder; Organiz. Materials = Organization of Materials; P- = Parent-rated; PEDsQL = Pediatric Quality of Life Inventory; Post = Post-test; Pre = Pre-test; Psy.soc. Hlth. = Psychosocial Health Summary Score; Punish. Sens. = Punishment Sensitivity; Reward Respons. = Reward Responsiveness; SPSRQ = Sensitivity to Punishment and Sensitivity to Reward Questionnaire for children; SSRT = Stop Signal Reaction Time; T- = Teacher-rated; **Bold + italic formatted number** = more than 30% responders; **Bold formatted number** = more than 50% responders; Children were classified as responders based on reliable change indices [98], [99].

## Discussion

The aim of this study was to determine the short- and long-term effects of a gamified training intervention (BGB) that targets multiple EFs (visuospatial WM, response inhibition and cognitive flexibility) compared to a placebo version of the intervention on various outcome measures in children with ADHD combined-type. In addition, to determine the unique effect of the inhibition and cognitive flexibility training tasks, we compared a full-active condition (where WM, inhibition, and cognitive flexibility were all in training-mode) to a partially-active condition (where only inhibition and cognitive flexibility were in training-mode).

Results indicated that only children in the full-active condition showed improvement on measures of visuospatial STM and WM. Inhibitory performance and interference control only improved in the full-active condition and the partially-active condition. However, no Treatment-condition x Time interactions (with or without Bonferroni corrections) were found for cognitive flexibility, verbal STM and WM, non-verbal complex reasoning, or child-rated psychosocial health, nor for any parent- or teacher-rated ADHD symptoms, EF behaviors, motivational behaviors, or general problem behaviors. Nonetheless, almost all measures showed significant Time-effects, including the teacher-ratings (effect sizes ranged from medium to large).

These findings suggest that improvements on inhibition and visuospatial STM and WM were specifically related to the type of treatment received. However, improvements on untrained EFs and behavior (*far transfer* effects) were mostly nonspecific (i.e., only interference control improved exclusively in the two conditions where EFs were trained). As such, in this multiple EF training, mainly nonspecific treatment factors—as opposed to the specific effects of training EFs—seem related to the far transfer effects on EF and behavior.

In many ways our findings are similar to those of previous placebo controlled (single) EF training studies in children with ADHD [21], [84], [85], [86], [23] (but note that only one of these studies [21] corrected for multiple testing). Most of these studies find differential treatment effects on outcome measures of trained EFs (although Kray et al. [23], as in the present study, found no significant differences on cognitive flexibility). However, such *near transfer* effects may not be surprising since many of these outcome measures are very similar to the training tasks themselves and improvement may be the result of a learned strategy instead of improved cognitive capacity [87]. Further, in most placebo controlled studies differential *far transfer* to untrained EF tasks has been limited, and differential effects on parent- or teacher-rated behavior (e.g., ADHD or EF) are generally not found. Only Klingberg et al. [86] found a differential effect of WM training on parent-rated ADHD. However, the placebo condition used in Klingberg et al. was considerably shorter in time than the training condition. This suggests a difference in parent involvement between the conditions, which may have interacted with the outcome of parent-rated ADHD behavior (e.g., through expectancy effects or inequality of parent-child interactions; see [15]). Another notable feature of the study of Klingberg et al. is that they did not include children with comorbid ODD. However, including ODD diagnosis as a covariate did not change the pattern of our main results. Therefore, the absence of comorbid ODD in the Klingberg et al. study seems an unlikely explanation for their distinctive findings on parent-rated ADHD. This assumption is further substantiated by the findings we presented in Table A (see S2 Appendix): Irrespective of treatment condition, children with comorbid ODD were at least as likely to improve on parent-rated ADHD behavior as children without comorbid ODD.

There are also several important differences between our findings and the findings of previous placebo controlled EF training studies. Although we used more stringent compliance criteria than most previous studies (i.e., completing 100% of the training sessions versus completing 80% of the training sessions), in our study only 3% of the participants failed to meet compliance criteria, whereas in previous studies 15–23% failed to meet compliance criteria. Since most previous studies also used an external reward system, a structured schedule for implementing the intervention, weekly contact with a coach, and performance feedback during training, the most obvious reason for this difference in compliance is the relatively strong gamification of BGB. This hypothesis is consistent with previous findings of increased time-on-training when EF training was gamified [45] (also see [40]), and with the finding that gaming increases the release of striatal dopamine [47], [48], which is associated with increased motivation to continue playing and performing [50].

Moreover, in contrast to the previous placebo-controlled studies, we found a significant improvement on teacher-rated ADHD behavior (effect sizes ranged from medium to large). Although this improvement was unrelated to specific effects of the EF training (as it was also found in the placebo condition), it is still a remarkable finding. Some have argued that EF training studies only find Time effects on parent-ratings but not on teacher-ratings because teachers, in contrast to parents, are only minimally involved in training and thus may be less biased than parents (e.g., by their expectancies of the training outcome) [31]. This suggests that generalization of improvement to teacher-ratings might represent relatively unbiased evidence of treatment induced changes in the child’s behavior. Nonetheless, it is unclear what caused this improvement. It seems unrelated to specific EF training effects, and the only nonspecific treatment factor that clearly distinguishes our study from previous studies appears to be the use of relatively strong gamification (i.e., teachers were not more involved than in previous studies). Is it possible that gamification somehow improved classroom behavior? For example, there is evidence that video game playing can enhance various cognitive skills (e.g., attention; see [88]). However, if playing video games by itself would be sufficient to improve classroom functioning in children with ADHD, it seems illogical that the participants in our study, who play commercial video games for 10 hours per week (see Table 1), did not improve before. Nonetheless, it may be that parents’ positive attitude towards this particular game enhanced its positive effects. For example, sharing the joy of achievement in the game with his/her parents could have enhanced the child’s appraisal of the game’s positive feedback and its effect on his/her self-esteem beyond that of commercial video games (as many parents don’t encourage children to indulge in commercial gaming). Although there is a link between parental praise and children’s self-esteem [89], and self-esteem has been found to mediate the relationship between ADHD and classroom functioning [90], future research should investigate this further. Furthermore, the gamification of BGB may also have impacted classroom functioning by enhancing children’s motivation to comply with treatment. If children were more motivated to comply with treatment than in other EF training studies, which is consistent with the relatively high compliance rate in our study, there may have been less need for parents to discipline their children during training. Evidence suggests that decreased negative parental discipline mediates the effect of ADHD treatment (e.g., medication and behavior therapy) on teacher-rated ADHD behavior [91]. Future EF training studies should use larger samples and appropriate process measures to further investigate these potential mechanisms of mediation.

Although some previous EF training studies in children with ADHD have found differential effects on interference control [23], [30] ([85] and [86] also found differential effects on the STROOP, but they only used the incongruent trials as outcome measure; baseline response times to congruent trials were not controlled for, making it impossible to calculate the interference score), our study is the first to find differential effects on response inhibition. In contrast to the placebo condition, response inhibition was improved in both the full-active condition and the partially-active condition, but no differences were found between these two experimental conditions. This suggests that a combined inhibition and cognitive flexibility training by itself (i.e., without WM) is sufficient to improve response inhibition in children with ADHD. Possibly, previous EF training studies investigating effects on measures of response inhibition in children with ADHD [29], [30], [32] found no improvements because their intervention did not include an inhibition training task (i.e., Hoekzema et al. [32] trained WM, cognitive flexibility, attention, planning and problem solving), or because their inhibition training task was based on a less appropriate response inhibition paradigm; the go/no-go task instead of the stop task [29], [30]. In contrast to the stoptask, the go/no-go task has been criticized as not functionally isolating inhibition (e.g., because of its interaction with selective attention and decision making, and the confounding effects of its prepotent response processes; see [2], [92], [93]). Nonetheless, since we did not investigate effects of the inhibition- and cognitive flexibility training separately, we can only speculate that the improvement on response inhibition was the result of our stop-task-based inhibition training. Additional research is needed to investigate this in more detail.

In contrast to our findings on other near transfer measures, no differential effects of EF training were found on the cognitive flexibility measure (neither with or without Bonferroni correction). This may be the result of the difference between the switch-cost (the index of cognitive flexibility) that was trained, and the switch-cost that was used as outcome measure of cognitive flexibility. Our outcome measure (the scaled contrast score on the TMT) measures *global* switch-cost (i.e., the difference between a block of switch-trials and blocks of non-switch trials), whereas the cognitive flexibility training focused on training *local* switch-cost (i.e., the difference between switch-trials and non-switch trials within a block of trials). Although, both types of switch-cost are considered valid measures of cognitive flexibility, evidence suggests that they tap somewhat different cognitive processes and can be differentiated on a neural level [94], [95]. Therefore, it could be argued that our outcome measure of cognitive flexibility was in fact a measure of far transfer. Future studies should investigate this further using more varied measures of cognitive flexibility.

The fact that far transfer was also found in the placebo condition might not (only) be explained by nonspecific treatment effects (e.g., effects of expectancies, self-fulfilling prophecies, attribution, gamification, or improved parent-child interactions), but may be the result of actual cognitive training in the placebo condition. Although the cognitive load in our placebo condition was very low, it could be argued that the requisite of the placebo tasks to focus attention for a substantial amount of time was sufficient to improve cognitive control (e.g., attention) and the behavior of our participants. However, this appears inconsistent with the very limited improvement on EF performance in the placebo condition, and the lack of effects resulting from other activities that require prolonged focused attention (e.g., paying attention in school, playing [educational] video games).

Because no wait-list control condition was utilized, it is not possible to determine to which extent our findings relate to effects of multiple testing, the passage of time, or (nonspecific) treatment factors. However, a previous study investigating BGB [31] found no improvement on parent- and teacher-rated ADHD and EF behavior in a wait-list control group, whilst they did find improvement in the group that was trained. This suggests that the current findings on ADHD and EF behavior are probably not attributable to mere passage of time or multiple testing (for a study of children with autism spectrum disorder see [100]).

In this study different EFs were trained simultaneously within the same training session. However, based on the current state of the literature it is unclear if this is indeed the best strategy for multiple EF training (i.e., there are no studies that directly investigate this). One could assume that training different EFs simultaneously is more effective (especially for transfer to daily life) than training one EF at a time (i.e., training each EF in separate sessions), because functioning in daily life also requires the use of multiple EFs at once. However, our results do not suggest that training three EFs per session (i.e., the full-active condition) has more effect on daily functioning than training two EFs per session (i.e. the partially-active condition). Future studies should further investigate this.

In the current study, far transfer effects were mostly nonspecific. However, we only investigated overall group differences (i.e., disregarding potential subpopulations that show differential responses to treatment), and children were allocated to treatment conditions irrespective of their individual EF deficits. Therefore, before discarding EF training as potential treatment for children with ADHD, future studies should examine moderators (e.g., severity of EF deficits; teacher expectancies) and mediators of treatment success (e.g., improvement on EF performance; parental praise), and should investigate effects of individually tailored EF training (i.e., to make optimal use of the available training-time future studies should match training focus to the specific EF problems of each individual child). Furthermore, to increase chances of finding far transfer that results from EF training specifically, training tasks should be made more ecologically valid (e.g., by using EF training tasks that resemble the complexity of problematic situations in daily-life) and should be intertwined with relevant real-life EF-taxing activities (e.g., completing chores in daily-life could be an additional goal in the EF training; for more suggestions see [96]). Finally, the domains of far transfer that were investigated in this study were limited to direct measures of performance and indirect measures of behavior (e.g., behavior as rated by parents, teachers or children). Future studies should also include direct measures of behavior. For example, a recent placebo-controlled WM training study [84] found no specific treatment effects on parent-rated behavior (teacher-rated behavior was not investigated), but found specific effects on aspects of experimenter-observed off-task behavior during an academic task.

In conclusion, our findings suggest that improvements on inhibition and visuospatial STM and WM were specifically related to the type of treatment received. However, improvements on untrained EFs and behavior were mostly nonspecific. As such, in this multiple EF training (BGB), mainly nonspecific treatment factors—as opposed to the specific effects of training EFs—seem related to the far transfer effects on EF and behavior.

## Supporting Information

S1 CONSORT Checklist CONSORT checklist Dovis et al. (DOC)

S1 Appendix Document containing Appendix 1 (including Fig A.) Appendix 1—Fig A The reward system: an overview of the medals and reward ribbons and the map with all the stickers (photograph courtesy of Arnold Brakenhoff). (DOCX)

S2 Appendix Document containing Appendix 2 (including Table A.). (DOCX)

S1 Dataset (SAV)

S1 Protocol Trial protocol 2011-DP-1526 Dovis et al. with attachm. (PDF)
