# Supplementary material for: Improving Executive Functioning in Children with ADHD: Training Multiple Executive Functions within the Context of a Computer Game. A Randomized Double-Blind Placebo Controlled Trial
Source: PLoS One. 2015 Apr 6;10(4):e0121651. doi: 10.1371/journal.pone.0121651 (PMC4386826; doi:10.1371/journal.pone.0121651)
Supplement: S1 Appendix — (DOCX) [file pone.0121651.s002.docx]

**Appendix 1**

Sebastiaan Dovis, Saskia Van der Oord, Reinout W. Wiers, and Pier J. M. Prins

**The External Reward System**

The standardized external reward system was the same for all participants. Its procedure was as follows:


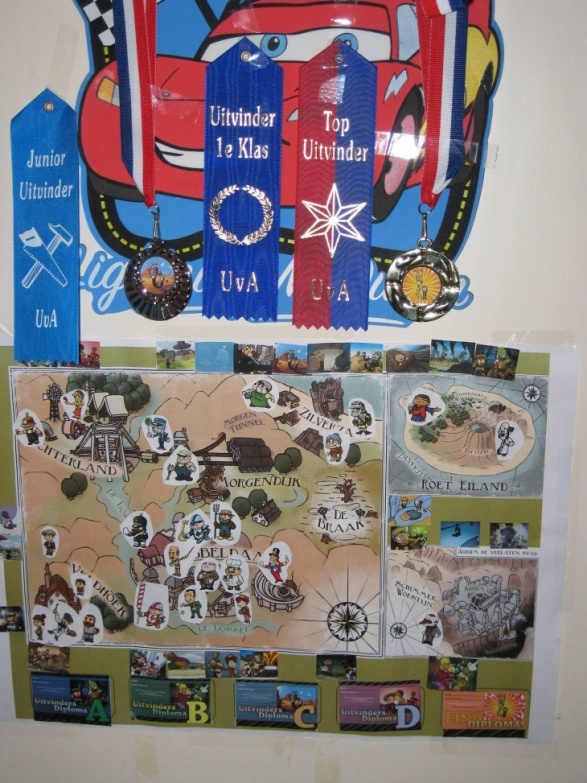
Children received a poster representing a map of all the areas in the game, and parents received 25 numbered envelopes (the numbers on the envelopes corresponded to the numbers of the training sessions). Parents were instructed to give the child an envelope after each completed training session (regardless of previous arguments or bad behavior). Each envelope contained two numbered stickers that could be pasted on corresponding numbers on the map. One sticker represented a game character that the child befriended in the training session, and the other sticker represented one of the inventions that was created in the training session. In this way the poster was used as an external representation of training achievement (see Fig. A). Every 5^th^ envelope contained two extra rewards: a medal or reward ribbon and a certificate-sticker (that could be pasted on the poster). These extra rewards were used to emphasize the completion of each of the five training weeks.

Fig. A The reward system: an overview of the medals and reward ribbons and the map with all the stickers (photograph courtesy of Arnold Brakenhoff)
