# Supplementary material for: Improving Executive Functioning in Children with ADHD: Training Multiple Executive Functions within the Context of a Computer Game. A Randomized Double-Blind Placebo Controlled Trial
Source: PLoS One. 2015 Apr 6;10(4):e0121651. doi: 10.1371/journal.pone.0121651 (PMC4386826; doi:10.1371/journal.pone.0121651)
Supplement: S2 Appendix — (DOCX) [file pone.0121651.s003.docx]

**Appendix 2**

Sebastiaan Dovis, Saskia Van der Oord, Reinout W. Wiers, and Pier J. M. Prins

**Table A.**

*Proportion of children with and without a diagnosis of ODD (according to the PDISC-IV) in each treatment group that show improvement on performance measures and rating-scales (i.e., responders)*

*Note.* BRIEF = Behavior Rating Inventory of Executive Function; CBTT = Corsi Block Tapping Task; CD = conduct disorder; DBDRS = Disruptive Behavior Disorder Rating Scale; FU = Follow-up-test (after 3 months); HSQ = Home Situations Questionnaire; Imp/Fun Seeking = Impulisivity/Fun Seeking; ODD = oppositional defiant disorder; Organiz. Materials = Organization of Materials; P- = Parent-rated; PDISC-IV = Diagnostic Interview Schedule for Children, parent version; PEDsQL = Pediatric Quality of Life Inventory; Post = Post-test; Pre = Pre-test; Psy.soc. Hlth. = Psychosocial Health Summary Score; Punish. Sens. = Punishment Sensitivity; Reward Respons. = Reward Responsiveness; SPSRQ = Sensitivity to Punishment and Sensitivity to Reward Questionnaire for children; SSRT = Stop Signal Reaction Time; T- = Teacher-rated; **Bold number** = more than 30% responders; **Bold + underlined number** = more than 50% responders; Children were classified as responders based on reliable change indices [98], [99]
