# Supplementary material for: Improving Executive Functioning in Children with ADHD: Training Multiple Executive Functions within the Context of a Computer Game. A Randomized Double-Blind Placebo Controlled Trial
Source: PLoS One. 2015 Apr 6;10(4):e0121651. doi: 10.1371/journal.pone.0121651 (PMC4386826; doi:10.1371/journal.pone.0121651)
Supplement: S1 Protocol — (PDF) [file pone.0121651.s005.pdf]

General Section

Project title

Improving Executive Functioning in Children with ADHD: Training Executive Functions within the Context of a Computer Game

Responsible researcher (if a PhD-project is concerned list the professor involved)

Sebastiaan Dovis, Pier Prins, Saskia van der Oord

Who conducts the research? (PhD-students, students, etc.)

Sebastiaan Dovis (PhD-student)

Responsible Department or Section

Developmental Psychology

Research location

Children are recruited from multiple outpatient mental-healthcare centers

Brief project description (max. 200 words)

Theories of ADHD suggest that deficits in executive functioning and motivation are at the core of the ADHD-syndrome, and play a pivotal role in explaining the problems children with ADHD encounter in daily life. Working memory, response inhibition and Cognitive flexibility are executive functions (EFs) that seem especially impaired in children with ADHD.

Research suggests that training the working memory of children with ADHD can improve their executive functioning, complex reasoning, and (parent-rated) ADHD symptoms. Moreover, these effects appear to persist until 3-6 months after training. Such long-term post-treatment effects are very important for clinical practice, especcially since the effect of a evidence based intervention such as medication, lasts only several hours or days.

In the present study we investigate the effects (e.g., on measures of EF, ADHD behavior, and general problem behavior) of a training intervention that not only aims to improve working memory, but also trains other EFs that are impaired in children with ADHD (response inhibition and cogntive flexibility). In addition, we account for the motivational deficits of children with ADHD by presenting the training within the context of a 3D computer game. After a intake session and a pre-test session children are trained for 25 sessions of 40 minutes, on a home-based computer for a period of 5 weeks. Training is followed one week later by a post-test session, and 3-months later by a follow-up session. In this study we examine three variants of the EF training.

1. *A full-active condition:* In this condition the working memory, inhibition and cognitive-flexibility tasks are all in training-mode. Training-mode entails that the difficulty level of the training task is automatically adjusted to the child's level of performance.
2. *A Partially-active condition:* In this condition the inhibition and cognitive-flexibility tasks are in training-mode, and the working memory task is in non-adaptive-mode. Non-adaptive-mode entails that the difficulty level is low and not adjusted to the child's level of performance (e.g., no more than two items have to be remembered).
3. A Non-adaptive (*placebo-like) condition.* In this condition the working memory, inhibition and cognitive-flexibility tasks are all in non-adaptive-mode.

Parents are informed about these three conditions and that they will be randomly assigned to one of these conditions (parents can not choose their condition; and after randomization they are not told to which condition they are assigned)

A comparable study using the same training (Braingame Brian) is now conducted in children with autism by Marieke de Vries (PhD-student in the department of Brain & Cognition), and has been approved by the Medical Ethics Committee of the Academic Medical Center (AMC).

Expected duration of the project

18 months

Expected number of participants

90 children *(*aged *8-12* years*)* with ADHD

Content Section

A1. When classifying the research as Medical vs. Non-medical, does it comply with A1, meaning it can be listed under category D (see also Appendix 1, 2.4)?

Yes, it falls into category D

A2. Are consenting adults selected, as described in A2?

Yes, indicate how according to A2

Answer:

Participant are recruited within a institutional environment (e)

A3. Are participants free to decide to participate and to stop for whatever reason, as listed under A3?

Yes

A4. Are participants subjected to a screening procedure to reduce the risks for adverse effects, as listed under A4?

Yes, because

Comment:

Children are screened on IQ and parent- and teacher-rated symptoms of ADHD, ODD, and CD. Parents are interviewed for symptoms of ADHD, ODD and CD. Children with ADHD are, only if they meet inclusion criteria, randomly assigned to one of the treatment conditions.

A5. Is there a risk for chance incidents that should be reported to the participant, as listed under A5?

No, the method precludes chance incidents

A6. Are participants fully informed before participating, and do they sign a consent form, as listed under A6?

Yes, please submit the information letter and the consent form as attachment

A7. Is participant privacy and anonymity guaranteed, as listed under A7?

Yes

A8. In case of deception, does the procedure comply with the conditions listed under A8? (full disclosure concerning risks, accurate debriefing)?

There is no deception

A9. Is there a risk that a substantial number of participants will drop out because the research is considered to be discomforting, as listed under A9?

No

B1. Does the research **fully** comply with the guidelines for Standard Research?

Yes, specify below and upload a concise research description (max 1 A4)

Answer:

B2. Standard research, department of Developmental Psychology

Appendix 1: Detailed Research Proposal

Workingtitel: Improving Executive Functioning in Children with ADHD: Training Executive Functions within the Context of a Computer Game
Projectnummer EC:

(v.1.1; 28 januari 2011)

Auteur:
Drs. S. Dovis

Promotores:
Prof. Dr. P.J.M. Prins
Prof. Dr. R.W.H.J. Wiers

Co-promotor:
Dr. S. van der Oord

Promovendus:
Drs. S. Dovis

Introduction

Child focused, evidence-based psychosocial interventions for children with ADHD are lacking (Landelijke Stuurgroep Multidisciplinaire Richtlijnen in de GGZ, 2005). A recent theory of ADHD proposes that self-regulation deficits - related to executive functions (EFs) such as working memory (WM) , response inhibition, and set shifting (Nigg, 2006) - are at the core of the ADHD-syndrome (Barkley, 1997).

Attempts have been made to train working memory deficits in children with ADHD through computerized training. Klingberg et al. (2005; see also Klingberg et al., 2002) successfully trained working memory (WM) in children with ADHD, using a computerized WM training, consisting of 25 days of training. At post-test, children performed significantly better on a visuo-spatial WM task, but also on tasks assessing verbal WM, response inhibition, and complex reasoning. Moreover, effects generalized to ADHD-behaviors and were maintained at three month follow-up. More recently Holmes et al. (2009) showed, in a sample of ADHD children, that a 5 week WM training had a stronger effect on the performance of non-trained WM tasks than stimulant medication. This effect was maintained at 6 months follow-up (for a review on WM training see Klingberg, 2010).

Unfortunately, there are fewer attempts to train other EFs that are impaired in children with ADHD (response inhibition and set-shifting). We are not aware of studies that train response inhibition in children with ADHD (for a training study in normal developing pre-school children see Thorell et al., 2009), and we know only one study were set-shifting is trained. In this study White and Shaw (2006) trained set-shifting in adults with- and without ADHD in two 30-minute sessions. They found that even this short training significantly improved set-shifting (both on trained and non-trained tasks) in both ADHD and non-ADHD training groups. Thus, although there is evidence that training WM can have a positive effect on children with ADHD, there are virtually no studies that investigate the training of response inhibition or set-shifting in children with ADHD.

It has been repeatedly observed by parents, teachers and clinicians that children with ADHD, can sustain attention, concentrate for longer periods of time and behave less impulsively when playing a computer game, (Barkley, 2006). This would imply that adding game elements (e.g., animation, storyline, sound effects, etc.) to a standard computerized WM-training would enhance its effects. We examined this additional value of gaming elements in a computerized WM training in children with ADHD and found that a WM training with game elements had a significantly greater impact on (a) children's motivation, (b) training performance and (c) posttraining WM-score compared to a WM-training without such game elements (Prins et al., 2010). The present project extends this pilot research and the current EF training studies in children with ADHD. Therefore, in this study WM, set-shifting and response inhibition will be trained within the context of a computer game.

The first aim of the present study is to examine whether training response inhibition and set-shifting can improve performance on non-trained inhibition and set-shifting tasks, can have a spill-over effect to untrained EFs and complex reasoning (as was found for WM in Klingberg et al., 2005), and whether the improvement of these EFs is associated with a reduction of ADHD symptoms, ADHD related behavior, and EF related problem behavior, and to what extend these effects are maintained at 3 month follow-up. The second aim of this study is to investigate the effect of adding a WM training to the response inhibition and set-shifting training. This is done to compare the relative (additional) impact of a visuospatial WM training to that of the inhibition and set-shifting training.

To investigate this we will randomly assign ADHD children to one of three training conditions: (1) a full and adaptive (A) training condition, where both WM, inhibition and set shifting are adaptively trained (Full A-training condition). Adaptive means that the difficulty level of the training adapts to the performance of the participant. (2) a training condition where response inhibition (inh) and set-shifting (ss) are adaptively trained and the WM training is presented non-adaptively and on a low initial training level (inh/ss A-training condition). And (3) a training condition where WM, response inhibition and set-shifting are presented non-adaptively (NA) and on a low initial training level (a placebo-like Full NA-training condition).

The effect of the interventions will be measured in five different domains: 1) EF performance and behavior (visuospatial and verbal WM, response inhibition, set-shifting, visuospatial and verbal short term memory, inhibition of interference, and an EF behavior questionnaire), 2) Complex reasoning, 3) ADHD characteristics, 4) General problem behavior, and 5) Motivation.

After a short ADHD screening, children will be tested at four different occasions, 1) T1.1 3 to 4 weeks before the intervention an intake session and the first part of testing will be done, 2) T1.2 1 to 3 weeks before the intervention the second part of testing will be done, 3) T2 1 week after the intervention a post-test session will be done, 4) T3 3 month follow-up after the intervention (see procedure).

We expect that the full A-training will have a stronger effect on EF performance and behavior, complex reasoning, ADHD characteristics, general problem behavior and motivation than the two other conditions at T2 and T3, and that the inh/ss A-training condition will have a stronger effect on all these measures compared to the Full NA-training condition at T2 and T3 (White & Shaw, 2006; Klingberg et al., 2005). Finally, we expect that the effects of training will remain significant at the three month follow-up (Klingberg et al., 2005).

Method

Participants

Ninety children aged 8 to 12 years with a diagnosis of ADHD combined-type participate: Children are recruited from outpatient mental-healthcare centers.

Children meet the following criteria: (a) a prior DSM-IV-TR (American Psychiatric Association, 2000) diagnosis of ADHD combined-type by a child psychologist or psychiatrist, (b) a score within the clinical problem range (95th to

100th percentile) on the ADHD scales of both the parent and teacher version of the Disruptive Behavior Disorder Rating Scale (DBDRS; Pelham, Gnagy, Greenslade, & Milich, 1992; Dutch translation Oosterlaan, Scheres, Antrop, Roeyers, & Sergeant, 2000), (c) meeting criteria for ADHD combined-type on the ADHD section of the Diagnostic Interview Schedule for Children for DSM-IV, parent version (PDISC-IV; Shaffer, Fisher, Lucas, Dulcan, & Schwab-Stone, 2000). The PDISC-IV is a structured diagnostic interview based on the DSM-IV, with adequate psychometric properties. (d) absence of Conduct Disorder (CD) based on the CD sections of the PDISC-IV, (e) absence of a prior DSM-IV-TR diagnosis of any autism spectrum disorder (ASD) according to a child psychologist or psychiatrist. (f) an IQ score ≥80 as measured by the short version of the Dutch Wechsler Intelligence Scale for Children (WISC-III; Kort et al., 2002). Two WISC-III subtests, Vocabulary and Block Design are administered to estimate Full Scale IQ (FSIQ). This composite score has satisfactory reliability (r =0.91) and correlates highly with FSIQ (r = 0.86; Sattler, 2001), (g) absence of any neurological disorder, non-verbal learning disorder (Nigg, 2006), or sensory (color blindness and vision) or motor impairment as stated by the parents, and (h) not taking any medication other than methylphenidate (children have to be able to discontinue medication at least 24 hours before each test session, allowing a complete wash-out; Greenhill, 1998).

Measures

1) **Behavior Rating Inventory of Executive Function questionnaire (BRIEF)** (Gioia et al., 2000; Smidts & Huizinga, 2009). The Dutch version of the BRIEF will be used to assess parent’s ratings of executive functioning of the participant. The questionnaire consists of 75 questions which have to be filled in by parents on a three point Likert scale (never, sometimes, or often). The questionnaire includes eight sub domains of executive function: Inhibit, Shift, Emotional Control, Initiate, Working Memory, Plan/Organize, Organization of Materials, and Monitor. The outcome measures will be the total score and the scores on the subscales Working Memory and Shift, as these are the EF functions targeted in the training.
2) **Reward and punishment sensitivity:** BIS/BAS Questionnaire (Colder & O`Conner, 2004). The Children’s version of the BIS/BAS measures sensitivity to reward and punishment. It contains 33 items and is divided in a BIS scale (15

items measuring sensitivity to punishment) and three subdimensions of the BAS scale: Drive (4 items), Reward Responsiveness (7 items), and Impulsivity/Fun Seeking (7 items). Each item is scored on a 5-point Likert scale (1=strongly disagree, 5=strongly agree). The questionnaire was translated to Dutch using a two-way translation procedure by an official translator and native English speaker (Luman et al., in prep). The predictor measurement will be the scores on the BIS and on the BAS scale.

3) **QoL**: Pediatric Quality of Life Inventory (PedsQL) (Varni et al., 2001; Bastiaansen et al., 2004). This questionnaire consists of 23 items, scored on a five-point Likert-scale ('never a problem' to 'almost always a problem'). The questionnaire is divided in four subscales: physical, emotional, social, and school functioning. The PedsQL was translated into Dutch using a two-way translation procedure (Bastiaansen et al., 2004). The outcome measures will be the total score and the scores on the four subscales.
4) **Stop task (inhibition)** (Logan, 1997; Morein-Zamir et al., 2008). In this task the participant has to respond to a picture of an arrow appearing on the computer screen as fast as possible by pressing a target key. When the arrow turns red however, the participant has to inhibit this response. In 25% of trials the arrow turns red. The timing or delay between the go and stop signals will be adjusted by the computer program for every participant using a tracking mechanism. At first the stop signal (the dog turning red) will appear after 300 ms. Following a successful stop, the delay will be lengthened by 50 ms, making the next stop trial harder to perform. After a failed stop, the delay will be shortened by 50 ms, making the next stop trial easier. The time needed to stop a response is measured for every participant as the main outcome measure: the stop-signal-reaction-time. Also the RT on go trials and the number of different errors (pressing the wrong button or failing to inhibit a response) will be outcome measures.
5) **Disruptive Behavior Disorder Rating Scale (DBDRS).** A Dutch digital version American Disruptive Behaviour Disorder Rating Scale is used in this study. The DBDRS is used to measure to what extent symptoms of disruptive behaviour disorders and of attention deficit/ hyperactivity disorder (ADHD) in children are present. Two versions of the DBDRS are used, respectively the parent and teacher version. The questionnaire consists of four subscales, respectively 42 items: Attention Deficit, Hyperactivity and Impulsivity, Oppositional Defiant Disorder

(ODD) and Conduct Disorder (CD). The items are scored on a four point Likert scale, ranging from 0 (“not at all”) to 3 (“lots”) to what extent the statement relates to the child. An example of an item is: “Often intentionally offends others”. Scores are calculated using raw subscale scores. The DBDRS shows reasonable reliability.

6) **Corsi Block Tapping Task forward and backward (spatial STM & WM)** (Corsi, 1972; Lezak, 1995, Berch, 1998). This task is a measure of the capacity of the visuospatial working memory in children. The CBTT consists of a wooden white board with nine, blue wooden blocks of 3.2 cm (Berch, Krikorian, & Huha, 1998). Children have to reproduce sequences of blocks. These sequences of blocks are shown to them by the researcher, who presents the sequence by tapping on the blocks with his finger. Whenever one of three responses of a certain sequence is accurate, the sequence is extended by one block. After three inaccurate, consecutive responses, the task is finished. The CBTT shows a good reliability. However, the reliability depends on the test form that is used (Schellig, 1997).
7) **The Trail Making Task (set-shifting).** Timed task that requires the individual to connect a series of letters and numbers in ascending order while alternating between numbers and letters.
8) **Digit recall forward and backward (verbal STM & WM).** The subject repeats a series of digits in the same or reverse of the order that they were presented.
9) **Raven coloured progressive matrices (complex reasoning).** Raven’s coloured progressive matrices (CPM) measures clear-thinking ability and is designed for young children ages 5-11 years and older adults. The test consists of 36 items in 3 sets (A, Ab, B), with 12 items per set. Administration time: 15-30 minutes.
10) **The Home Situation Questionnaire (HSQ)** (Barkley, 1990; Dutch translation Prins) is designed to assess the impact of attention problems at home and in public situations (e.g., mealtimes, in the car).

EF training

The computer game “Brain Game Brain” is developed by a Task Force of clinical child (neuro-)psychologists at the University of Amsterdam in close collaboration with a professional game developer, Shosho (www.shosho.com). The 25 session game and train EF-training will be available for research February 1st, 2011. The EF training is embedded in a fantasy world with a main character with which the player – the

child with ADHD - can identify. Throughout the game, the main character may walk through the gameworld and has to perform various assignments, which are in part fun games and in part EF-training tasks (see below). Play time and training time are balanced within each training session. The adaptive versions of the training tasks have several levels of difficulty which allow the child to make progress at his own level. In these adaptive tasks the level of difficulty systematically increases with each training session, in a way that is similar for all children. The level of difficulty in a particular session can be adjusted upward or downward, if the child does not reach the minimum set level or performs very well. In the non-adaptive version of the training tasks start at a low difficulty level (for WM) or practice level (for inhibition and set-shifting) and do not adapt to individual performance during training (see below for a description for each training task). The computer game has been developed in such a way that the three EFs, i.e.,WM, Inhibition, and set-shifting, can be trained separately. For example, a player can train low and non-adaptively on the WM-tasks, while the Inhibition and set-shifting-training tasks are trained adaptively.

Children will play and train at home over a period of 5 weeks, for a total of 25 sessions of about 45 minutes each. In each session EFs are trained during 30 minutes (10 minutes per EF) and the child has at least 15 minutes to explore the game world. In an introductory session the child and his parent will be introduced to the game. The child will get the opportunity to try out the game. Rules of the game are explained. Every week during training, the parents and child will be called by the research assistant to monitor adherence to the training protocol and to discuss and solve problems.

EF-training tasks

Three Executive Functioning Tasks have been developed for the present project: a working memory training, an inhibition training (inhibitory motor control) and a set shifting training (Task Force ADHD & Computer, 2008). The three tasks are built into a computer game. The EF training tasks can be set to a higher and adaptive difficulty level or to a low non-adaptive (placebo-like) difficulty level.

<u>Adaptive Working Memory training</u> – The visuospatial WM-training consists of training three WM components: short-term memory, updating, and holding information on-line (Baddeley, 2000; Miyake & Shah, 1999). These components will

be trained in 5 tasks, which are variations on the 4x4 grid (16 squares) where squares light up and form a sequence which has to be reproduced by the child (Prins et al., 2010).

Low and non-adaptive Working Memory training – The low and non-adaptive visuospatial WM-training consists of the first of the five working memory training tasks and is presented on a low difficulty level. The difficulty level of this task does not change during the 5 week training (is non-adaptive).

Adaptive inhibition training - The inhibition training is based on the Stop Signal Task (Logan. 1994).which trains the child to inhibit a behavioral impulse (behavioral inhibition). The Stop Signal Task generates an objective measure of inhibition, the Stop Signal Reaction Time (SSRT), which is generally lower in children with ADHD than in normal controls (Huizinga et al., 2008). The training aims to improve the SSRT. During the training, the child has to react to a stimulus in 75% of the trials. In the other 25%, just after the stimulus is shown and the child has started his reaction, a stop-signal appears and the child has to inhibit his response. The time interval between stimulus and stop signal gradually shortens as the child progresses in the training, which taxes the child’s inhibitory power.

Low and non-adaptive inhibition training - During the low and non-adaptive inhibition training, the child has to react to a stimulus in 100% of the trials (there is no stop-signal; which makes the task less difficult) and the difficulty level of the low training version of the tasks remains the same throughout the whole training.

Adaptive set-shifting training - The set-shifting training trains the child to suppress a response and at the same time activates an alternative, more appropriate response (switching). The training is based on the Switch Task (see King et al., 2007). ‘Switchcost’ is used to measure the ability to switch between two competing responses, and is the extra time it costs to switch between two responses relative to continuing with the same behavior. On every trial during the training the child gets visual feedback on his reaction times (switches and non-switches). The level of difficulty gradually increases during the training.

Low and non-adaptive set-shifting training – The low and non-adaptive set-shifting training trains the child to respond correctly to two stimuli (the child has to press left or right), the child does not have to switch between two sets of stimuli (only non-

switch trials are presented), and the difficulty level of the low training version of the tasks remains the same throughout the whole training.

Procedure

The study procedure consists of eight steps of contact between the experimenter and the participant and/or his parents:

1. First contact; information and intake by telephone.

Parents receive a letter with information about the training study from the mental-health care centers with a request to participate in our study. If the parents and children are interested to participate they can contact the research assistants. The research assistant will than provide the parents with information about the study and its procedure, ask about some inclusion criteria (e.g. use of medication, willingness to discontinue medication during test sessions, intentions to start with other forms of treatment during the course of the study, problems with task performance), and obtains demographic characteristics of parents and child (e.g. address, telephone numbers, email, teachers name, name of the school). The assistant will than ask parents to fill in an online screener and asks them for permission to contact the child's teacher for information.

2. ADHD online screening (parent and teacher).

After filling in a digital informed consent (appendix 2), parents complete an online version of the DBDRS (ADHD screener). Also, the teacher is contacted by the research assistant, gets informed about the research and the training and is asked to fill in the online DBDRS. If the child meets the inclusion criteria of the DBDRS, he and his parents are invited to the first intake session.

3. First intake session and first part of testing

This session takes place at the mental-health care institution. During this session two experimenters are present. After a short introduction, parents are presented with a paper version of the informed consent (appendix 2) and are asked some remaining intake questions (e.g. on game experience). Next, the parents stay in the room with one experimenter to be interviewed with the PDISC-IV and the child go's with the other experimenter to another room for testing. During testing the IQ measure (WISC-

III subtests) is administered to the child. Testing is intermitted by a 5 minute break. If the child meets the inclusion criteria of the PDISC-IV and the WISC-III, he and his parents are invited to the second intake session and to take part in the training, and he will be randomly assigned to one of the three aforementioned training conditions. Parents will also be asked to keep medication use as equal as possible during the course of the study. Parents will be asked to report any changes in medication use and other forms of treatment so that we can covariate for these possible confounding variables in our analyses.

<u>4. Second part of testing and estimation of training level</u>

This session takes place at the mental-health care institution. During this 90 minute session one experimenter is present. After a short introduction parents are asked to fill in questionnaires in the waiting-room (the BRIEF, the HSQ, the BIS/BAS, and a motivational measure). The child stays in the room with the experimenter and the visuospatial short term memory (STM; CBTT forward) and WM task (CBTT backward), the verbal STM (digit recall forward) and WM task (digit recall backward), the inhibition tasks (Stop signal task and the STROOP), the set-shifting task (the Trail Making Task), and the complex reasoning task (Raven coloured progressive matrices) are administered to the child. Testing is intermitted by a 5 minute break.

<u>5. Parents and children collect the training computer and receive instructions</u>

This session takes place at university. During this 30 minute session one experimenter is present. Parents and child come to the university to collect the training computer and rewards (e.g. medals to keep children motivated) and get a detailed instruction about the training and its procedure. After this session parents and child go home and start with the training.

<u>6. Contact by telephone about the progress in the training.</u>

The children will perform the training for a fixed duration of 45 minutes each session (10 minutes for each EF training task and 15 minutes for exploring in the game). The total number of training sessions is 25. Children are asked to play the game 4-5 times a week for a period of 5 to 6 successive weeks, until they finish all sessions. The training is embedded in the computer game. Every week during training a research

assistant will call the parents and child to see how the training is going. The research assistant will advise parents and child, help them to deal with training related problems and encourage parents and child to continue with the training. Finally, the research assistant will check and note if there are any changes in medication use or in other forms of treatment.

7. Post-measurement directly after training.

This session takes place at the mental-health care institution. During this 90 minute session one experimenter is present. Parents can bring the training computer back to the institution or can choose to bring it back to the university. After a short introduction parents are asked to fill in questionnaires in the waiting-room (the DBDRS, the BRIEF, the HSQ, the BIS/BAS, and a motivational measure). The child stays in the room with the experimenter and the visuospatial short term memory (STM; CBTT forward) and WM task (CBTT backward), the verbal STM (digit recall forward) and WM task (digit recall backward), the inhibition tasks (Stop signal task and the STROOP), the set-shifting task (the Trail Making Task) and the complex reasoning task (RAVEN) are administered to the child. Testing is intermitted by a 5 minute break. Also, the teacher is contacted again and is asked to fill in the online DBDRS. Finally, the 3 month follow-up appointment is made with the parents.

8. Follow-up 3 months after training

This final session takes place at the mental-health care institution. During this 90 minute session one experimenter is present. After a short introduction parents are asked to fill in questionnaires in the waiting-room (the DBDRS, the BRIEF, the HSQ, the BIS/BAS, and a motivational measure). The child stays in the room with the experimenter and the visuospatial short term memory (STM; CBTT forward) and WM tasks (CBTT backward), the verbal STM (digit recall forward) and WM task (digit recall backward), the inhibition tasks (Stop signal task and the STROOP), the set-shifting task (the Trail Making Task) and the complex reasoning task (RAVEN) are administered to the child. Testing is intermitted by a 5 minute break. Also, the teacher is contacted again and is asked to fill in the online DBDRS. Finally, we thank the parents and child for their participation.

Statistical Analysis

First, analyses of variance will be conducted to test for group differences at pretreatment (T1). Chi-square tests will be used for categorical variables. Second, training effects will be examined with 3 (type of intervention: full A-training, inh/ss A-training, full NA-training) by 3 (time: test sessions T1, T2, and T3) repeated measures ANOVA's. Effect sizes (η^2) will be reported for all analyses. Using Cohen's (1988) guidelines, effect sizes smaller than 0.06 are considered small, effect sizes between 0.06 and 0.14 are considered medium, and effect sizes above 0.14 are considered large.

Based on the studies of Klingberg et al. (2005) and Prins et al. (2008) we estimate that there will be a medium effect size for the primary outcome measures (EF measures, AD/HD symptoms). Power analysis shows that to detect an overall difference of a medium effect size between the three conditions, 102 participants are needed (power=.8, alpha=.05). Post-hoc comparisons between the three conditions, detecting this medium effect, a difference between working memory training and cognitive flexibility training, requires 34 participants per condition (power=.7, alpha=.05, one-tailed). Therefore, 102 participants (34 per condition) need to participate.

Budget

Traininglaptops + muizen en verzekering	6 x 900 / 2	=	2700
Testlaptops	2 x 400	=	800
Laptoptassen	6 x 50 / 2	=	150
Beloningsysteem			
Medailles	200 x1,50	=	300
Vaantjes	300 x 1	=	300
Stickers		=	50
Enveloppen		=	50
Diploma's		=	50
Telefoonkosten		=	50
Versnaperingen		=	50
Reiskosten		=	150
Benzinekosten		=	200
			___________+
**Totaal (incl. voorschot)**			**4850 euro**

Planning ADHD training studie 2011-2013

2011

Januari

- kiezen meettaken en vragenlijsten
- Onderzoeksvoorstel schrijven + opsturen naar ethische commissie.
- materiaal bepalen en geld aanvragen bij bestuur (o.a. 7 laptops, medailles, vaantjes, telefoonkosten, benzinekosten en overige kosten)
- requirements doc. schrijven voor aanpassingen schaakbordtaak en gameversie
- Req. Doc schrijven voor aanpassing SOPT + stand alone maken
- Stoptaak van geluid voorzien
- projectaanvraag TOP laten ondertekenen
- Contact opnemen en afspraken maken instellingen
- Studenten werven
- Onderzoek indienen bij Trial register.

Februari

- Meettaken en vragenlijsten aanpassen en compleet maken
- Studenten selecteren en inwerken + onderz voorstel laten schrijven
- Ruimte voor studenten regelen
- Contact instellingen leggen en info praatjes geven aan medewerkers
- Laptops aanschaffen
- Handleiding training schrijven en beloningsysteem maken

Maart

- Meettaken en vragenlijsten compleet
- Instellingen langs voor werving en voorlichting

- Eerste brieven de deur uit bij 3 instellingen
- Inplannen eerste afspraken
- Studenten inwerken voor testen

April
- Begin april eerste 30 intake afspraken (voor eerste 20 kinderen)
- Eerste 20 experimentele afspraken
- Bij instellingen langs voor werving en voorlichting
- Begin eerste trainingen (eind april)

Mei (mei vakantie: 30 april – 8 mei)
- Bij instellingen langs voor werving en voorlichting
- Telefonisch contact onderhouden met lopende trainingen
- Student werven BRIEF, WG, motivatie studie data analyse

Juni
- Einde training eerste cohort
- Nameting eerste cohort
- Studenten regelen voor na de vakantie
- 3 nieuwe instellingen geregeld voor na de vakantie

Juli (zomervakantie 23 juli – 4 sept)
- Einde eerste training eerste cohort
- Nametingen eerste cohort
- Inwerken studenten voor na de vakantie
- Brieven versturen bij 3 instellingen voor cohort in september (1ste week juli)
- Intake en experimentele afspraken maken met deelnemers cohort 2 voor september

Augustus (VAKANTIE)
- Voorbereiden nieuw cohort september

September
- Bij instellingen langs voor werving en voorlichting
- tweede cohort 30 intake afspraken (voor 2e cohort van 20 kinderen)
- Nieuwe studenten werven voor cohort 2 en 3
- Follow-up 1ste cohort
- 20 experimentele afspraken cohort 2
- Brieven de deur uit bij 3 nieuwe instellingen voor cohort 3

Oktober (herfstvakantie: 15 tot 23 oktober)
- Begin training cohort 2
- Intake cohort 3

November
- Brieven de deur uit 3 nieuwe instellingen cohort 4
- Einde training cohort 2
- Nameting cohort 2
- Begin training cohort 3

December
- Intake cohort 4

2012
Januari
- Brieven de deur uit cohort 5 bij 3 nieuwe instellingen
- Einde training cohort 3
- Nameting cohort 3
- Begin training cohort 4
- Intake cohort 5

Februari
- Follow up cohort 2
- Intake cohort 5
- Einde training cohort 4
- Nameting cohort 4

Maart
- Einde training cohort 4
- Nameting cohort 4
- Begin training cohort 5

April
- Follow-up cohort 3
- Einde training cohort 5
- Nameting cohort 5

Mei
- Einde training cohort 5
- Nameting cohort 5
- Follow-up cohort 4

Juni
- Follow up cohort 4

Juli
- Follow-up cohort 5 (eind juli)

Augustus (VAKANTIE)
- Follow-up cohort 5

September – december
- Uitloop

Appendix 2. Informed Consent

Titel onderzoek: *'het trainen van zelfcontrole bij kinderen met ADHD'*

Toestemmingsverklaring medewerking onderzoek

Hierbij geef ik ______________________________ (naam en voorletter(s) ouders)

ouder/voogd van ______________________________ (naam en voorletter(s) kind)

Geboren op ______________________________ (geboortedatum kind)

□ **WEL/GEEN*** **toestemming** voor medewerking aan het onderzoek "*het trainen van zelfcontrole bij kinderen met ADHD*" (* doorhalen wat niet van toepassing is).

Ik verklaar hierbij op voor mij duidelijke wijze te zijn ingelicht over de aard, methode en doel van dit onderzoek. De schriftelijke informatie, behorend bij deze verklaring, is mij overhandigd. Mijn vragen zijn naar tevredenheid beantwoord. Ik stem geheel vrijwillig in met deelname aan dit onderzoek. Ik behoud daarbij het recht deze instemming weer in te trekken zonder dat ik daarvoor een reden hoef op te geven. Indien mijn onderzoeksresultaten gebruikt zullen worden in wetenschappelijke publicaties, dan wel op een andere manier openbaar worden gemaakt, zal dit volledig geanonimiseerd gebeuren. Mijn persoonsgegevens zullen niet door derden worden ingezien zonder mijn uitdrukkelijke toestemming.

Adres ______________________________

Postcode en Woonplaats ______________________________

Datum ______________________________

Handtekening ______________________________

Als ik nog verdere informatie over het onderzoek zou willen krijgen, nu of in de toekomst, kan ik me wenden tot de onderzoeker Drs. Sebastiaan Dovis (tel: 020-5256298 of e-mail S.Dovis@uva.nl, Roetersstraat 15, 1018 WB Amsterdam, kamer A7.24. Voor eventuele klachten over dit onderzoek kunt u zich wenden tot het lid van de Commissie Ethiek van de afdeling Psychologie van de Universiteit van Amsterdam, de heer Dr. Wery van den Wildenberg (tel: 020-5256686; e-mail W.P.M.vandenWildenberg@uva.nl, Roetersstraat 15, 1018 WB Amsterdam, kamer A7.10).

Appendix 3: Information Letter for Parents

"Onderzoek naar het trainen van executieve functies bij kinderen met ADHD"

Geachte deelnemer aan het onderzoek "executieve functie training bij kinderen met ADHD":

De GGZ instelling van uw kind verleent medewerking aan het onderzoek "executieve functie training bij kinderen met ADHD" van de Universiteit van Amsterdam (afdeling ontwikkelingspsychologie). Over het onderzoek wordt u hieronder nader geïnformeerd. Het betreft onderzoek waarvoor de deelname van kinderen uit een instelling als de uwe onontbeerlijk is. In het algemeen vinden de kinderen het leuk om aan dergelijk onderzoek mee te doen.

Voordat het onderzoek begint, is het belangrijk dat u kennis neemt van de procedure die in dit onderzoek wordt gevolgd. Leest u derhalve het onderstaande s.v.p. zorgvuldig door.

Doel van het onderzoek

Recente theorieën van ADHD gaan ervan uit dat de problemen die kinderen met ADHD in hun dagelijkse leven ondervinden te herleiden zijn tot tekorten in hun executieve functioneren en tot motivationele afwijkingen. Werkgeheugen, Inhibitie en Set-shifting zijn de executieve functies (EFs) die bij kinderen met ADHD het meest beperkt zijn.

Onderzoek laat zien dat het trainen van het Werkgeheugen van kinderen met ADHD zorgt voor een sterke verbetering van het executieve functioneren, het complex redeneren en voor een significante vermindering van de ADHD symptomen. Bovendien blijken deze effecten 3-6 maanden na de training nog aanwezig te zijn. Een dergelijk langdurig effect na de behandeling is voor de klinische praktijk van zeer grote waarde; vooral omdat het effect van bijv. medicatie enkele uren tot dagen na gebruik geheel verdwenen is.

In de huidige studie onderzoeken we de effecten (o.a. op EF maten en ADHD symptomen en gedragsproblemen) van een training waarin niet alleen het Werkgeheugen, maar ook de andere EFs waarop kinderen met ADHD beperkt zijn worden getraind (Inhibitie en Set-shifting). Daarnaast houden we in deze training rekening met de motivationele afwijkingen van kinderen met ADHD door de training aan te bieden in de vorm van een 3D computerspel.

Na een intake sessie van en een voormeting van beide 90 minuten trainen kinderen 25 sessies 40 minuten lang achter de computer thuis gedurende 5 weken. Vervolgens vindt er een nameting plaats en volgt er na 3 maanden een follow-up meting.

We testen in dit onderzoek de werking van drie varianten van de EF training.

1. Een variant waarin alle EF trainingen op een hoog niveau worden aangeboden.
2. Een variant waarin alle EF trainingen op een zeer laag niveau worden aangeboden.
3. Een variant waarin een deel van de EF trainingen op een hoog niveau en een deel van de EF trainingen op een zeer laag niveau worden aangeboden.

Om psychologische effecten zoveel mogelijk te voorkomen kunnen wij u vooraf niet aangeven welke variant van de training uw zoon of dochter aangeboden krijgt. Na afloop van de training wordt u uiteraard volledig op de hoogte gesteld van de trainingsvariant die uw zoon of dochter heeft uitgevoerd. De training is nieuw en wij kunnen dus niet garanderen dat zij effect heeft.

Instructie en procedure

Het onderzoek bestaat vijf sessies/afspraken en 25 trainingsessies. Tijdens de eerste afspraak (intake) nemen we bij u interview af waarin we vragen stellen over het gedrag van uw kind en nemen we bij uw kind een aantal taakjes af om te bepalen of hij mee kan doen aan de training. Deze afspraak duurt, inclusief pauzes ongeveer 90 minuten. Tijdens de tweede afspraak (voormeting) vragen we u om een paar vragenlijsten in te vullen en nemen we bij uw kind een aantal computertaken af. Deze afspraak duurt ongeveer 90 minuten. Tijdens de derde afspraak krijgen u en uw kind uitleg over de computertraining en kunt een trainingscomputer mee naar huis nemen en kunt u de training beginnen. Tijdens de training wordt u eens per week gebeld door een van de onderzoeksassistenten om u te assisteren bij eventuele problemen of vragen. De vierde afspraak vindt een week nadat de training is afgerond plaats. Tijdens deze afspraak vinden de nametingen plaats en wordt u weer gevraagd om een aantal vragenlijsten in te vullen en doet uw kind een aantal taakjes. Deze afspraak duurt opnieuw ongeveer 90 minuten. Om te bepalen hoe de eventuele effecten van de training stand houden volgt er 3 maanden na afloop van de training nog een follow-up afspraak die hetzelfde verloopt als de vierde afspraak en wederom 90 minuten duurt.

De tests of taakjes die wij bij uw kind afnemen zijn niet bijzonder moeilijk of vermoeiend. Het kind heeft hiervoor verder geen ervaring met computers nodig. Het kind krijgt voor de test nog een uitgebreide instructie en voldoende oefening, zodat zij/hij de test goed zal begrijpen en goed zal kunnen uitvoeren. Omdat voorkennis over het onderzoek de uitkomsten van het onderzoek kunnen vertekenen kunnen we verder geen inhoudelijke mededelingen doen over de taken. Zoals gezegd wordt u wordt na afloop op de hoogte gesteld van de uitkomsten van het onderzoek.

Vrijwilligheid

Als uw kind niet aan het onderzoek wil meedoen, of als u niet wilt dat uw kind aan het onderzoek deelneemt, dan wordt uw kind niet in het onderzoek opgenomen. Als uw kind gaandeweg besluit dat zij/hij wil stoppen, dan kan dat op elk moment, zonder opgaaf van redenen en zonder dat dit op enige wijze gevolgen voor uw kind zal hebben. Tevens kunt u tot 24 uur na dit onderzoek alsnog uw toestemming om gebruik te maken van de gegevens van uw kind intrekken. Mocht uw kind haar/zijn medewerking staken, of mocht u binnen 24 uur uw toestemming intrekken, dan zullen de gegevens van uw kind worden verwijderd uit onze bestanden en vernietigd.

Ongemak, Risico's en Verzekering

Uit ervaring met voorgaande, vergelijkbare onderzoeken is gebleken dat er van enig ongemak voor de deelnemers niet of nauwelijks sprake is. Omdat dit onderzoek geen risico's voor de gezondheid of veiligheid van het kind met zich mee brengt, is geen speciale verzekering afgesloten.

Vertrouwelijkheid van onderzoeksgegevens

De gegevens van dit onderzoek zullen door de onderzoekers alleen worden gebruikt voor nadere analyse en voor eventuele publicatie in wetenschappelijke tijdschriften. Hierbij wordt geen gebruik gemaakt van de persoonsgegevens van het kind, en blijft de anonimiteit van het kind gewaarborgd.

Vergoeding

Kinderen krijgen tijdens en na afloop van het onderzoek een medaille voor deelname, verder zijn er geen vergoedingen mogelijk.

Nadere inlichtingen

Mocht u vragen hebben over dit onderzoek, vooraf, tijdens of achteraf, dan kunt u zich wenden tot de verantwoordelijke onderzoeker, Drs. Sebastiaan Dovis (tel: 020-5256298 of e-mail S.Dovis@uva.nl, Roetersstraat 15, 1018 WB Amsterdam, kamer A724. Voor eventuele klachten over dit onderzoek kunt u zich wenden tot het lid van de Commissie Ethiek van de afdeling Psychologie van de Universiteit van Amsterdam, de heer Dr. Wery van den Wildenberg (tel: 020-5256686; e-mail W.P.M.vandenWildenberg@uva.nl, Roetersstraat 15, 1018 WB Amsterdam, kamer A7.10).
